# Mirk/Dyrk1B controls ventral spinal cord development via Shh pathway

**DOI:** 10.1007/s00018-023-05097-9

**Published:** 2024-01-31

**Authors:** N. Kokkorakis, K. Douka, A. Nalmpanti, P. K. Politis, L. Zagoraiou, R. Matsas, M. Gaitanou

**Affiliations:** 1 Laboratory of Cellular and Molecular Neurobiology-Stem Cells, Hellenic Pasteur Institute, Athens, Greece; 2 Division of Animal and Human Physiology, Department of Biology, National and Kapodistrian University of Athens, Athens, Greece; 3 Athens International Master’s Programme in Neurosciences, Department of Biology, National and Kapodistrian University of Athens, Athens, Greece; 4 Center of Basic Research, Biomedical Research Foundation of the Academy of Athens, Athens, Greece; 5 School of Medicine, European University Cyprus, Nicosia, Cyprus

**Keywords:** Chick neural tube, Ventral progenitor domains, Neuronal subtypes, Apoptosis, Motor neurons, Dyrk1B

## Abstract

Cross-talk between Mirk/Dyrk1B kinase and Sonic hedgehog (Shh)/Gli pathway affects physiology and pathology. Here, we reveal a novel role for Dyrk1B in regulating ventral progenitor and neuron subtypes in the embryonic chick spinal cord (SC) via the Shh pathway. Using *in ovo gain-and-loss-of-function* approaches at E2, we report that Dyrk1B affects the proliferation and differentiation of neuronal progenitors at E4 and impacts on apoptosis specifically in the motor neuron (MN) domain. Especially, Dyrk1B overexpression decreases the numbers of ventral progenitors, MNs, and V2a interneurons, while the pharmacological inhibition of endogenous Dyrk1B kinase activity by AZ191 administration increases the numbers of ventral progenitors and MNs. Mechanistically, Dyrk1B overexpression suppresses Shh, Gli2 and Gli3 mRNA levels, while conversely, Shh, Gli2 and Gli3 transcription is increased in the presence of Dyrk1B inhibitor AZ191 or Smoothened agonist SAG. Most importantly, *in phenotype rescue* experiments, SAG restores the Dyrk1B-mediated dysregulation of ventral progenitors. Further at E6, Dyrk1B affects selectively the medial lateral motor neuron column (LMCm), consistent with the expression of Shh in this region. Collectively, these observations reveal a novel regulatory function of Dyrk1B kinase in suppressing the Shh/Gli pathway and thus affecting ventral subtypes in the developing spinal cord. These data render Dyrk1B a possible therapeutic target for motor neuron diseases.

**Supplementary Information:**

The online version contains supplementary material available at 10.1007/s00018-023-05097-9.

## Introduction

Spinal cord (SC) development is orchestrated by coordinated actions of morphogens such as Sonic hedgehog (Shh), bone morphogenetic protein (BMP) and Wnt molecules that act by forming gradients in opposite directions along the dorsoventral axis of the developing SC [1–5]. Shh is a key molecule for the specification of neuronal and glial cell lineages during SC development. Shh is produced initially by the notochord (NC), which acts as an organizing center, later during neurogenesis by the medial floor plate (MFP), and finally, during gliogenesis, by the lateral floor plate (LFP) [3, 6–9]. Shh regulates the combinatorial expression of a set of transcription factors that are necessary and sufficient to specify each neuronal subtype, resulting in the formation of five discrete ventral progenitor domains (p3, pMN, p2, p1, and p0) that are arrayed along the dorso-ventral axis of SC [2, 6]. In the chick SC, Shh controls cell cycle progression of neural progenitors and their survival by maintaining progenitor cell proliferation and preventing cell death during SC development [9–11].

Dyrk1B kinase (also referred to as MIRK; minibrain-related kinase) belongs to the DYRK kinase family (for dual-specificity tyrosine-(Y)-phosphorylation-regulated kinases) comprising proline-directed kinases that phosphorylate tyrosine, serine and threonine amino acid residues [12–17]. DYRKs acquire their catalytic activity during their translation by intramolecular auto-phosphorylation at the second tyrosine residue located at the conserved YxY motif of their activation loop [17]. In particular, the intramolecular auto-phosphorylation of DYRKs is supported by a characteristic sequence motif, the DYRK homology box (known as the DH box), which is located at the N-terminal of their catalytic domain [15, 17]. Dyrk1B is normally expressed at high levels in skeletal muscle and testis with increased relative expression in cardiac muscle and brain compared to other tissues [18–20]. During development Dyrk1B has a critical role in myogenesis [21–23], spermatogenesis [24] and adipogenesis [25–28], while it is implicated in human diseases, such as metabolic syndrome [25–28], and tumor progression promoting survival and chemoresistance in cancer cells [29–38]. Dyrk1B mainly acts as a negative regulator of cell cycle progression via destabilization of cyclin D1 [16, 20, 38–46] and as a prosurvival factor [20, 22, 29, 33, 41, 47–49]. Moreover, Dyrk1B acts as central mediator of Sonic hedgehog/Gli [44, 50–54], PI3K/mTOR/AKT [44, 52, 54] and RAF/MEK/ERK [41, 55] signaling pathways in development and disease.

In contrast to its extensively studied pathophysiological role in several organ systems, Dyrk1B function in the nervous system remains unexplored. Dyrk1B is closely related to the Dyrk1A gene, while minibrain (MNB) is their orthologous gene in Drosophila named according to the brain phenotype of mutant flies [15, 56, 57]. Mutation of MNB causes abnormal arrangement of neuroblasts in the larval brain, resulting in smaller optic lobes and brain hemispheres, suggesting that MNB is crucial for proper neuroblast proliferation during neurogenesis [15, 56, 57]. Notably, the minibrain phenotype in mutant flies is associated with behavioral deficits in learning, memory, visual and olfactory tasks [15, 56, 57]. Although the role of Dyrk1A in neurogenesis is well documented [57–63], the function of its closely related kinase, Dyrk1B, in CNS development remains elusive. In a previous study we obtained first evidence that Dyrk1B may be implicated in neurogenesis [42–44]. We have shown that Dyrk1B promotes cell cycle exit and neuronal differentiation in mouse neuroblastoma cells via cyclin D1 cytoplasmic relocation and proteasomal degradation [42, 43]. This function is counteracted by binding to the scaffolding protein RanBPM, which facilitates Dyrk1B proteasomal turnover [42]. These observations suggest that Dyrk1B may have a similar role in cell cycle progression/exit and differentiation of neural progenitors during neurogenesis in vivo [42–44]. To address this question, here we investigated the expression of Dyrk1B in the embryonic chick and mouse SC and used the early chick neural tube as a model system to explore the role of Dyrk1B in neurogenesis. We found that Dyrk1B is expressed in the notochord (NC) and floor plate (FP), as well as in cycling progenitors of the ventricular zone (VZ) and in neurons. Using *in ovo gain-and-loss-of-function* and *phenotype rescue* approaches in the E2 chick SC and subsequent analysis at E4 and E6 we uncovered a novel role for Dyrk1B kinase during spinal cord development, in controlling the numbers of ventral progenitor and neuron subtypes, as well as in the columnar organization of spinal motor neurons (MNs) via the Sonic hedgehog/Gli pathway.

## Materials and methods

### Cloning of chick Dyrk1B partial cDNA

To clone chick Dyrk1B cDNA we performed BLAST searches to identify EST clones corresponding to cDyrk1B using BBSRC chicken EST data (http://www.chick.manchester.ac.uk/).

We detected two EST clones: 603108058F1 cloneID = ‘ChEST50c16’ and 603209321F1 cloneID = ‘ChEST186i5’, but neither contained the whole reading frame nor the 3’-end of coding region of cDyrk1B that is highly different from the 3’-end of the coding region of its paralogue gene Dyrk1A. This was critical to distinguish Dyrk1B from Dyrk1A mRNA expression. Οpen reading frames of human, mouse and rat Dyrk1B orthologs display high similarity, as 92.64% between human and mouse, 93.17% between human and rat, and 96.72% between mouse and rat. Taking advantage of the high similarity between Dyrk1B coding regions among species, we then used primers corresponding to the 3’- end of mouse Dyrk1B coding region, in order to clone the 3’-end of chick Dyrk1B coding region by applying RT-PCR in total RNA isolated with Trizol from E4 chick SC. For cloning we used oligo dT and the primers FOR2D1B: 5’-CCCAAGCTTCCGTTGCCTTGGACGACC-3’ and REV2D1B: 5’- CCGGAATTCTCATGAGCTGGCTGCTGTGC-3’ and Q5 High-Fidelity DNA Polymerase (New England Biolabs, M0491). We cloned a cDNA fragment of 253 bp, which then was subcloned into the Hind III and EcoRI restriction sites of pBluescript II KS (–) (Stratagene) plasmid and then subjected to Sanger Sequencing. This revealed that the 253 bp cDNA fragment corresponds to the 3’-end of cDyrk1B coding region and displays 100% similarity with mouse, 96.05% with rat and 90.91% with human Dyrk1B, respectively (Fig.S1).

### Plasmids

For electroporation experiments, we used a bicistronic expression vector pCAG-mDyrk1B-IRES-NLS-GFP, co-expressing mDyrk1B coding region and nuclear targeted GFP. mDyrk1B coding region was obtained by applying PCR with Q5 High-Fidelity DNA Polymerase (New England Biolabs, M0491) in pCMV-SPORT6 plasmid containing mDyrk1B full length cDNA (clone: BC019545, IMAGE: 4511845) and primers D1BFOR1: 5’-CTAGCTAGCGCCACCATGGCCGTCCCACCA-3’ and D1BREV2: 5’-CCGATATCTCATGAGCTGGCTGCTGTGCTCTGG-3’. The cDNA fragment of 1890 bp that corresponds to the whole coding region of mDyrk1B, was then subcloned into Nhe I and EcoR V restriction sites of the pCAG-IRES-NLS-GFP empty vector. All clones were subjected to Sanger Sequencing analysis for verification. For in situ hybridization (ISH) experiments, we used a cDNA fragment of 253 bp corresponding to the 3’-coding region of cDyrk1B cDNA, a cDNA fragment of 396 bp corresponding to the 3’-coding region of mDyrk1B cDNA, both of them subcloned into the Hind III and EcoRI restriction sites of pBluescript II KS plasmid (Stratagene). For Sonic Hedgehog riboprobes, we cloned a cDNA fragment of 498 bp corresponding to the 5’-coding region of mSHH by applying PCR with Q5 High-Fidelity DNA Polymerase (New England Biolabs, M0491) in pCMV6-Entry plasmid, containing mShh coding region Myc-DDK-tagged (Mouse Sonic hedgehog NM_009170, ORIGENE, MR227201), and primers mSHHFOR: 5’-CCCAAGCTTGCCAGCGGCAGATAT-3’ and mSHHREV: 5’- CGGGAATTCGTCCTTCACCAGCTTG-3’. The PCR product of 498 bp, was then subcloned into the Hind III and EcoRI restriction sites of pBluescript II KS plasmid (Stratagene) and then subjected to Sanger Sequencing analysis for verification.

### Chicks

Chicken eggs, provided by a local supplier, were incubated horizontally at 37 °C, with > 60–90% humidity until the desired Hamburger and Hamilton (HH) developmental stages for *gain-and-loss-of-function* and *phenotype rescue* experiments*.*

### Mice

C57BL/6 J mice were handled in strict accordance with good animal practice, as defined by the relevant European and Greek animal welfare bodies, based on 3 + 1R: Replacement, Reduction, Refinement and Respect. Specifically, all procedures complied to the European and National Laws for Laboratory Animal Use (Directive 2010/63/EU and Greek Law, PD 56/2013), according to FELASA recommendations for euthanasia and the Guide for Care and Use of Laboratory Animals of the National Institutes of Health.

### Unilateral *in ovo* electroporation

*Gain-of-function* experiments *(GOF)* were performed by applying unilateral *in ovo* electroporation of pCAG-mDyrk1B-IRES-NLS-GFP or pCAG-IRES-NLS-GFP vector as control, in the developing chick neural tube as previously described [64–66]. Briefly, chick embryos at HH 12–14 (E2) were injected with plasmid DNA solution into the lumen of the central canal of the neural tube and then subjected to unilateral electroporation. Plasmids for electroporation were used at concentration of 2–2.5 µg/µl in TE (10 mM Tris–HCl, 1 mM EDTA, pH 7.5) containing 0.05% Fast Green (Sigma F7252). A few microliters of plasmid solution were injected with a glass microcapillary pipette into the lumen of the SC central canal by mouth pipetting. The microelectrodes made of platinum were spaced ~ 4 mm apart and gently placed in lateral position against the vitelline membrane surrounding the neural tube such that an applied electric current across the medio-lateral plane of the neural tube drove the negatively charged DNA into the cells on only the one side of the neural tube. Chick embryos were pulsed 5 times each for 50 ms with an interval of 950 ms at 25 V, by using a square wave electroporator (BTX Harvard Apparatus ECM 830). The embryos were harvested at E4 or at E6 and were prepared for immunohistochemistry (IHC), RNA in situ hybridization (ISH), Western blot analysis (WB) or qRT-PCR. For BrdU-labeling, chick embryos received 20 µl BrdU (1 mg/ml) on top of each embryo, dropwise with a pipette, 2 h before sacrifice.

### *Loss-of-function* (LOF) and *phenotype rescue in ovo* experiments

*Loss-of-function* (LOF) experiments, were performed by using the Dyrk1B-selective inhibitor AZ191 (Cayman No. 17693). Specifically, 600 µg of AZ191 previously dissolved in DMSO, were injected with an insulin 29G syringe, at E2 chick embryos at three points, with a 45-degree angle via three small holes made in the vitelline membranes, in such a way that the entire embryo was surrounded with the compound. AZ191 was injected in a total volume of 1 ml, by adding sterile PBS (without CaCl_2_ and MgCl_2_, Gibco, Cat No 10010-015), and supplemented with 1% (v/v) penicillin–streptomycin (SIGMA, P4333). Similarly, activation of Shh pathway was performed by using the Smoothened agonist, SAG (Abcam, ab142160). Specifically, 1 µg of SAG [67], previously dissolved in DMSO, was injected with an insulin 29G syringe, at E2 chick embryos as described above for AZ191. *Phenotype rescue* experiments were performed by administration of either AZ191 or SAG, 2 h after unilateral *in ovo* electroporation in order to minimize the stress inflicted on the embryos.

### Western blot (WB)

Chick and mouse brain and SC homogenates derived from several developmental stages, were prepared in a ratio 1:10 in ice cold lysis buffer containing: 10 mM Tris/HCl, pH 7.5, 150 mM NaCl, 1% Triton X-100, 1 mM EDTA, 1 mM EGTA, 0.1% SDS, 0.5% sodium deoxycholate, protease inhibitor cocktail set I (Calbiochem/Merck Cat No 539131) phosphatase inhibitors (PhosSTOP, Roche Life Science, Cat No 04906837 001). Tissues homogenates were lysed at 4 °C in a tube roller for 4–6 h. Then, homogenates were centrifuged at 1300×*g* at 4 °C for 5 min, and supernatants were collected. Protein concentration was estimated by a modified Lowry assay (DC Protein assay from Bio-Rad). Proteins were separated by 10% or 12% SDS-PAGE electrophoresis and transferred onto Porablot nitrocellulose (Macheray-Nagel, Cat No 741280). Blocking was performed in 5% non-fat milk in TBST containing 10 mM Tris–HCl pH 8.0, 150 mM NaCl, 0.1% Tween 20, for 1 h at room temperature (RT), followed by overnight incubation with primary antibodies. Incubation with appropriate HRP-conjugated secondary antibodies was performed for 2 h at RT. Primary and secondary antibodies were diluted in 2.5% non-fat milk in TBST and three washes were performed in TBST. Protein bands were detected by the enhanced chemiluminescence substrate (ECL) containing: 1.25 mM Luminol, 198 µM Coumaric acid, and 0.0096% H_2_O_2_ in 50 mM Tris–HCl pH 8.8. Densitometric analysis of protein bands and normalization relative to β-tubulin or GAPDH was performed using ImageJ software (NIH) (RRID:SCR_003070). All experiments were performed three times (n = 3), unless otherwise noted, by using protein lysates derived from pools of 3 animals corresponding to each experimental group. Quantitative data are mean values and error bars represent SEM.

### Tissue preparation

Chick and mouse embryos were harvested and fixed overnight at 4^0^ C with 4% paraformaldehyde (PFA) in phosphate buffered saline (PBS) pH 7.4. Adult pregnant mice were euthanized by isoflurane inhalation and perfused transcardially with PBS and then 4% PFA. Chick or mouse dissected SCs were then washed in PBS, cryoprotected for 16 h to 2 days at 4 °C with 20% sucrose in PBS for chick or 30% sucrose for mouse tissues, respectively, until osmotic equilibrium occurred. Tissues were then mounted in OCT (VWR Chemicals, Cat No 361603E), frozen and cryosectioned at 20 µm, and processed for immunostaining or RNA in situ hybridization (ISH). Serial sections placed on adjacent slides to allow for IHC or ISH at the same rostro-caudal level.

### Immunohistochemistry (IHC)

Cryosections of chick and mouse SC were subjected to antigen retrieval for heat-mediated epitope unmasking by incubating the slides in 10 mM Sodium citrate pH 6.0 for 30 min at 70 °C. After washing the slides 3 times for 10 min each, with 1X PBS, the slides of cryosections were blocked with 5% normal donkey serum (NDS) containing 0.1% v/v Triton X-100 in PBS for chick tissues or 0.2% v/v for mouse tissues respectively, for 1 h at RT. Primary antibodies were diluted in 2.5% NDS containing 0.05% v/v Triton X-100 in PBS, for chick tissues or 0.1% v/v for mouse tissues and tissue cryosections were incubated overnight at 4 °C, while cryosections were incubated with the appropriate secondary antibodies Alexa-Fluor 488, Alexa-Fluor 546 and Alexa-Fluor 647 (Invitrogen/ThermoFisher Scientific) for 2 h at RT. Cell nuclei labeled with TOPRO-3 and /or Hoechst (Molecular Probes, Invitrogen Cat No 33258). Slides were mounted with MOWIOL 488 Reagent (Calbiochem, Cat No 475904). Digital images were acquired by performing confocal microscopy using Leica TCS SP5II and Leica TCS SP8 confocal microscopes and images were analyzed using ImageJ software (NIH) (RRID:SCR_003070). Statistical analysis was performed by using at least n = 12 sections obtained from 4 animals corresponding to each experimental group. Normalization of cell numbers was performed to GFP^+^ cells or to total cell nuclei. Quantitative data were presented as mean values and error bars correspond to standard error of the mean (SEM).

### In situ hybridization (ISH)

Slides with 4% PFA-fixed cryosections were incubated with pre-hybridization buffer containing: 50% deionized formamide, 5X SSC buffer, 5X Denhardt’s solution (Invitrogen, Cat No 750018), 250 µg/ml yeast t-RNA (Roche Life Science, Cat No 10109223001), and 500 µg fish sperm DNA (Roche Life Science, Cat No 11467140001) in a humified chamber containing: 5X SSC for 3–4 h at 65 °C. Non-radioactive in situ hybridization (ISH) was performed in the hybridization buffer, which is same with the pre-hybridization buffer containing plus the DIG-labeled riboprobe. Hybridization buffer containing the DIG-labeled riboprobe before used was previously incubated at 85 °C for 5 min for denaturation of riboprobes. Preparations for DIG-labeled probes were carried out as previously described [64–66]. On the following day, slides were washed with 0.2X SSC for 2 × 30 min at 65 °C, 0.2X SSC for 5 min at RT, with B1 buffer (0.1 M Tris /HCl, pH 7.5 and 0.15 M NaCl) for 5 min at RT. Sections were blocked with B1 supplemented with 10% normal donkey serum (NDS) for 1 h, at RT, before incubating in an anti-DIG alkaline phosphatase (AP) conjugate (see Antibodies section) in B1 buffer supplemented with 1% NDS at 4 °C. On the following day, slides were washed with B1 buffer for 2X5 min at RT, with B3 buffer (0.1 M Tris/HCl, pH 7.5, 0.1 M NaCl, and 50 mM MgCl2) for 2X5 min at RT. Slides were then incubated with AP buffer (100 mM Tris /HCl, pH 9.5, 50 mM MgCl2, 100 mM NaCl, 0.1% Tween-20, and 1 mM levamisole) for 10 min at RT. Color reaction development started by adding AP buffer containing 10 µl/ml NBT/BCIP (Roche Life Science, Cat No 11681451001). The reaction was performed in the dark at RT for several hours to overnight. Color reaction was stopped by washing slides with PBS. After washing, sections were mounted in DPX mounting (Sigma-Aldrich, Cat No 06522). Digital images were acquired by performing transmitted light-bright field microscopy in ZEISS Axiovert 200 (ZEISS) and Leica TCS SP8 (LEICA microsystems) microscopes. Representative images from reproducible results were presented.

### Antibodies

All primary and secondary antibodies used are listed in Supplementary Table 1 and 2, respectively.

### RNA isolation, cDNA synthesis and real-time qRT-PCR

Total RNA was extracted from E4 chick SC, using Trizol Reagent (Ambion/Life Technologies, Cat No 15596-018) and then was incubated with DNase I (Takara Bio, Cat No 2270A). 1 µg of total RNA was used for first strand cDNA synthesis by using the ImProm-II Reverse Transcription System (Promega, Cat No A3800) and oligo dT primer, according to the manufacturer’s instructions. Real-time qRT-PCR analysis was carried out in a Mini-Opticon Real-Time PCR System (Bio-Rad, Cat No CFD-3120) using KAPA SYBR FAST qPCR Master Mix (Kapa Biosystems, KK4602) according to the manufacturer’s instructions. The analysis of relative gene expression was performed with 2^−ΔΔCt^ Method, by using the chick housekeeping gene glyceraldehyde-3-phosphate dehydrogenase (cGAPDH) as an internal reference. Real-time qRT-PCR analysis was carried out in three independent experiments (n = 3), by using mRNA pools of 3 chick embryos corresponding to each experimental group in technical triplicates. Relative gene expression data were presented as the mean of triplicate values obtained from three independent experiments. Error bars represent SEM. Primer sets used were: FOR cSHH: 5’-CAAAGCAGAAAACTCAGTGGCA-3’ and REV cSHH: 5’-TGACGTAGAAGAGCTTTCGGG-3’, FOR cGLI3: 5’-GCATGTGCCTTCTGCCTTATCTAG-3’ and REV cGLI3: 5’- GGTCAAAGCTGTGATCAGATAGGG-3’, FOR cGLI2: 5’-TGGCACAAGGAGTGCCGCAG-3’ and REV cGLI2: 5’-GTACGTCACGGGGTTGATGGG-3’ FOR cGAPDH: 5’-TCGGAGTCAACGGATTTGGC-3’ and REV cGAPDH: 5’-GCCCATTTGATGTTGCTGGG-3’.

### Statistical analysis

Before analysis, the normality of values was verified with the Shapiro–Wilk normality test using IBM SPSS Statistics for Windows Version 20.0 (RRID:SCR_019096). Statistical analysis was performed using two-tailed Unequal variance t test (Welch t test) to assess the significance of differences between two groups. The results are shown as mean values of experiments described above in each M&M section ± Standard error of the mean (SEM). Probability values p ≤ 0.05 were considered as statistically significant (*p ≤ 0.05; **p ≤ 0.01, ***p ≤ 0.001). All analyses were performed using Microsoft Excel 2013 (RRID:SCR_016137).

## Results

### Dyrk1B is expressed in the notochord, floor plate, proliferating progenitors in the ventricular zone and post-mitotic motor neurons in chick and mouse spinal cord

**Fig. 1:**
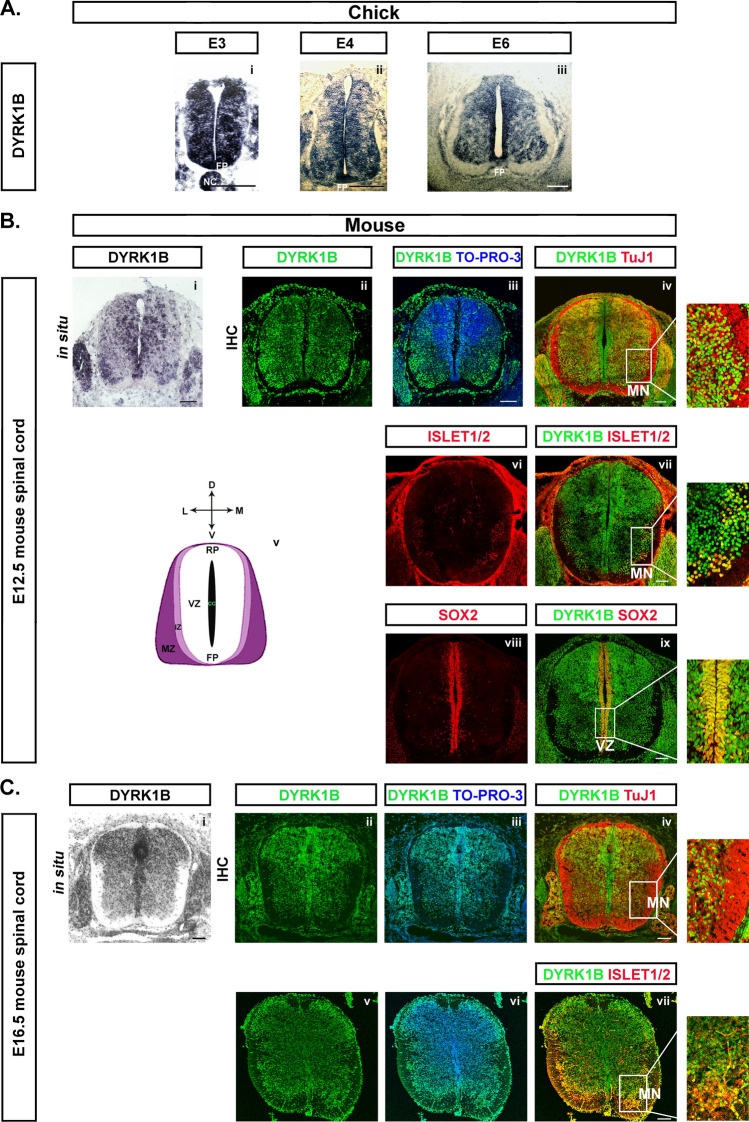
Dyrk1B expression in chick and mouse spinal cord. **A** Dyrk1B mRNA is mainly expressed in embryonic chick spinal cord by notochord (NC), floor plate (FP), cycling progenitors in the ventricular zone (VZ) and by motor neurons (MNs) in the mantle zone (MZ), as revealed by in situ hybridization (ISH) on transverse brachial spinal cord sections. The abundant Dyrk1B mRNA expression observed at E3 (i) is gradually restricted to the VZ area adjacent to the central canal (CC) and in the MN domain at E4 and E6 (ii–iii). **B** Similar Dyrk1B expression is observed in E12.5 mouse spinal cord (corresponding to E4 of chick) both at the mRNA (i) and protein levels (ii, iii), where Dyrk1B is expressed by TuJ1^+^ post-mitotic neurons of MZ (iv) and by Islet1/2^+^ MNs (vi, vii), as well as by Sox2^+^ cycling progenitors in VZ (viii, ix). Spinal cord domains are depicted in the scheme left (v). **C** At E16.5 mouse spinal cord, Dyrk1B is abundantly expressed in the grey matter and the VZ (i–vii), both at mRNA (i) and protein levels (ii–iii), Dyrk1B co-expression is shown in TuJ1^+^ post-mitotic neurons (iv) and Islet1/2^+^ MNs (vii). Scale bars: 100 µm. TO-PRO-3: nuclei staining

Τo establish the time-course of Dyrk1B expression in the developing chick and mouse CNS we performed Western blot analysis, using a specific monoclonal anti-Dyrk1B antibody on chick and mouse brain and SC protein lysates derived from different developmental stages, as indicated (Fig. S2). Dyrk1B protein expression was evident at all stages, declining over time. To further investigate Dyrk1B gene expression, we proceeded to RNA in situ hybridization (ISH) and immunohistochemical (IHC) analysis in transverse sections of chick and mouse SC using a 253 bp riboprobe corresponding to the 3’-end of cDyrk1B coding region that displays 100% identity with the corresponding 3’ region of mDyrk1B (Fig. S1). At early stages of chick spinal cord development (E3) Dyrk1B is ubiquitously expressed in neuroepithelial progenitors. At later stages (E4 and E6) though its expression is more evident in the floor plate (FP) and mantle zone (MZ) where neurons reside, as well as in the ventricular zone (VZ), where Dyrk1B is expressed in cycling neural progenitors (Fig. 1A). In mouse SC, Dyrk1B gene expression was analyzed at E12.5 and E16.5, by ISH using a 396 bp riboprobe corresponding to the 3’-end of mDyrk1B coding region, and by IHC at E12.5 and E16.5 (Fig. 1B, C). Again, at E9.5 Dyrk1B displays a broad expression throughout the SC (Fig. S3), while at E12.5 and E16.5, Dyrk1B expression is mainly detected in the FP, cycling Sox2^+^ progenitors of the VZ, TuJ1^+^ postmitotic neurons and in Islet1/2^+^ MNs (Fig. 1B, C).

### Dyrk1B overexpression promotes cell cycle exit and neuronal differentiation

To investigate the role of Dyrk1B in SC development, we applied unilateral *in ovo* electroporation at E2 chick SC in order to overexpress mDyrk1B, using the bicistronic expression vector pGAG-mDyrk1B-IRES-NLS-GFP (Dyrk1B/GFP embryos) or the control vector pGAG-IRES-NLS-GFP (GFP embryos), allowing both to use the GFP reporter gene as marker for transfected cells. 48 h after Dyrk1B/GFP electroporation, we confirmed a 3.0-fold increase in Dyrk1B mRNA and a 2.7-fold increase in protein levels (Fig. S4), respectively, by comparison to wild-type SC.

**Fig. 2:**
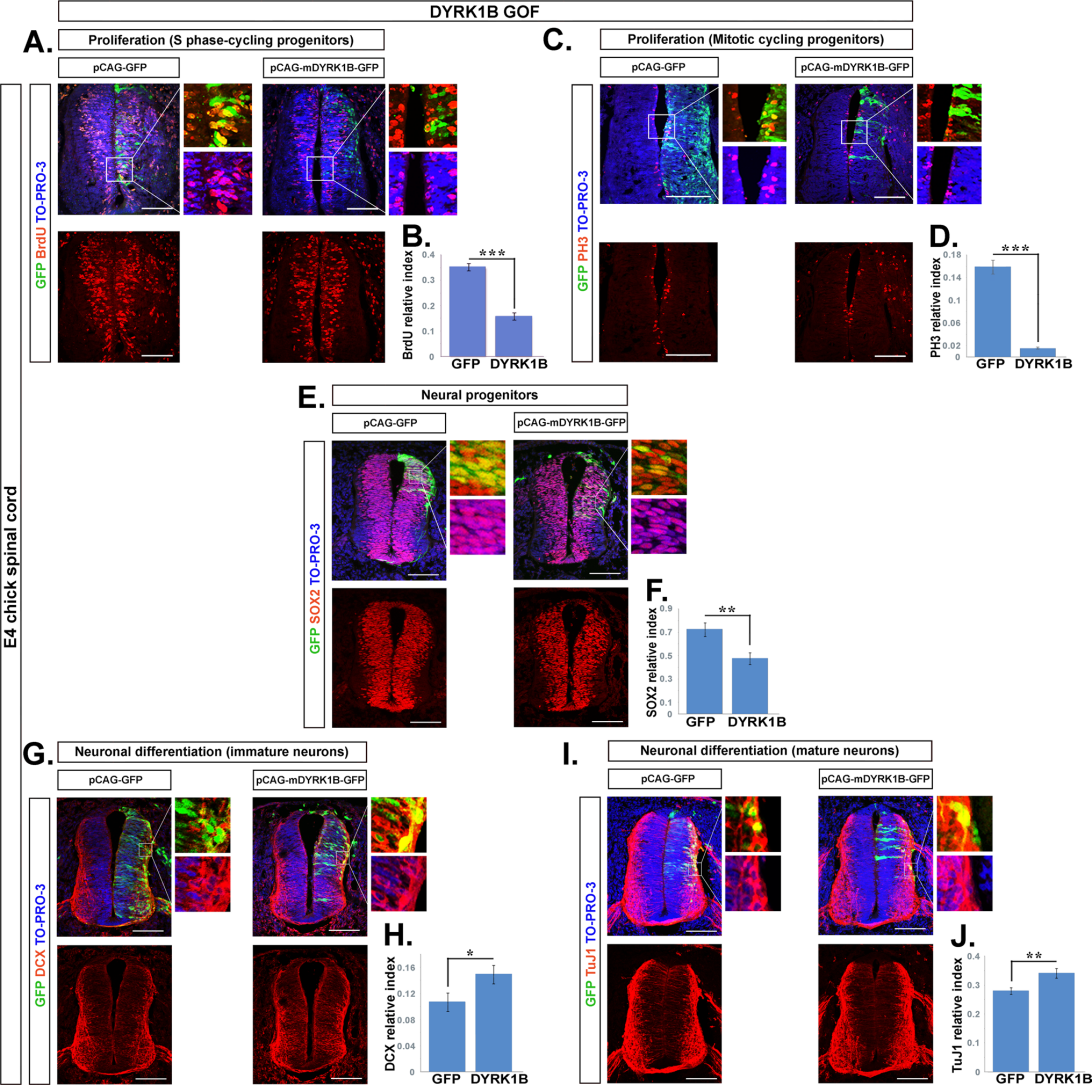
Dyrk1B overexpression at E2 promotes at E4 cell cycle exit and neuronal differentiation.** A**, **B** Dyrk1B^+^/GFP^+^ electroporated cycling progenitors co-expressing the S-phase marker BrdU were significantly reduced by 2.22 ± 0.20-fold (p ≤ 0.001, n = 18 sections from 5 embryos), compared to control. **C**,** D** Similarly, the relative index of Dyrk1B^+^/GFP^+^ cycling progenitors co-expressing the mitotic marker PH3 showed a dramatic decrease by 10.16 ± 0.98-fold (p ≤ 0.001, n = 18 sections from 5 embryos), compared to GFP^+^/PH3^+^ cells in control embryos. **E**, **F** Significant reduction by 1.53-fold ± 0.16 (p ≤ 0.01, n = 18 sections from 4 embryos) in the relative index of cells expressing the pluripotency marker Sox2, compared to control embryos. **G**, **H** The relative index of electroporated Dyrk1B^+^/GFP^+^ cells co-expressing Doublecortin (DCX) is increased by 1.39-fold ± 0.13 (p ≤ 0.05, n = 13 sections from 4 embryos) compared to GFP-electroporated embryos. **I**, **J** The relative index of electroporated Dyrk1B^+^/GFP^+^ cells co-expressing βΙΙΙ-tubulin (TuJ1) is increased by 1.22-fold ± 0.06 (p ≤ 0.01, n = 24 sections from 4 embryos) compared to GFP^+^ cells in control embryos**.** Analysis was performed by calculation of the relative index corresponding to the ratio of the number of double positive cells GFP^+^ /specific marker^+^ to total GFP^+^ cells. Scale bars: 100 µm. TO-PRO-3: nuclei staining. Data are mean ± SEM, *p ≤ 0.05, **p ≤ 0.01, ***p ≤ 0.001; ns, non-significant (two-tailed Unequal variance t test). Spinal cord sections correspond to the brachial level

Since Dyrk1B has been previously shown to act as a G0-checkpoint kinase that promotes cell cycle exit [20] and terminal differentiation of proliferating myoblasts [21–23], immature male germ cells [24], and mouse neuroblastoma cells [42], we investigated the effect of mDyrk1B overexpression on the cell cycle. To detect cycling progenitors in S-phase, we performed a 2-h pulse with the thymidine analog 5-Bromo-2´-Deoxyuridine (BrdU) before sacrifice of electroporated embryos, followed by IHC and confocal microscopy on transverse cryosections of SC at the brachial level. We observed a 2.22-fold ± 0.20 reduction (***p ≤ 0.001, n = 18 sections from 5 embryos) in the fraction of BrdU^+^/GFP^+^ cells over total GFP^+^ cells (relative index) in Dyrk1B/GFP embryos compared to control GFP embryos (Fig. 2A, B). Moreover, we noted a dramatic decrease by 10.16-fold ± 0.98 (***p ≤ 0.001, n = 18 sections from 5 embryos) in the relative index of phosphohistone H3 (PH3) mitotic cells (Fig. 2C, D), with a concomitant 1.53-fold ± 0.16 (**p ≤ 0.01, n = 18 sections from 4 embryos) decrease in the relative index of cells expressing the neural precursor marker Sox2 (Fig. 2E, F). Next we examined the effect of Dyrk1B overexpression in neuronal differentiation. We found that in Dyrk1B/GFP embryos the relative index of GFP^+^ cells co-expressing the early neuronal marker doublecortin (DCX) was increased by 1.39-fold ± 0.13 (*p ≤ 0.05, n = 13 sections from 4 embryos), as compared to GFP controls (Fig. 2G, H). Similarly, the index of the pan-neuronal marker βΙΙΙ-tubulin (TuJ1) cells was increased by 1.22-fold ± 0.06 (**p ≤ 0.01, n = 24 sections from 4 embryos), compared to GFP embryos (Fig. 2I, J). Thus, our data demonstrate that forced Dyrk1B expression promotes cell cycle exit and neuronal differentiation of proliferating precursors.

### Dyrk1B overexpression promotes apoptosis specifically in the motor neuron domain and decreases the number of motor neurons and V2a interneurons

**Fig. 3:**
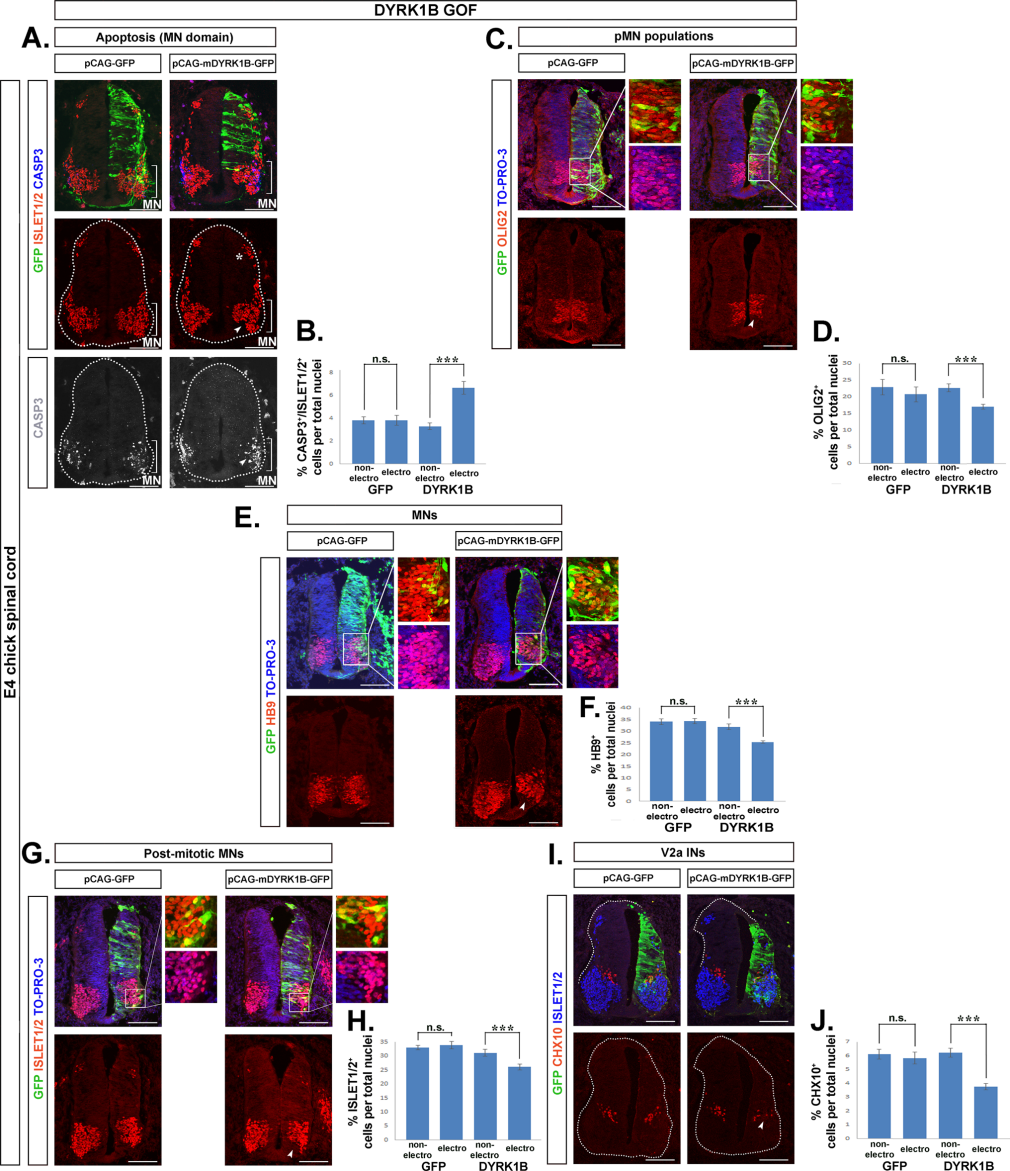
Dyrk1B overexpression promotes apoptosis in the motor neuron domain and decreases the number of pMNs, motor neurons and V2a interneurons. **A**, **B** The ratio of Casp3^+^/Islet1/2^+^ cells over total nuclei in the MN domain (arrowheads) is increased by 102.74% ± 17.02 (p ≤ 0.001, n = 12 sections from 4 embryos) in the Dyrk1B/GFP electroporated side of the SC when compared with the contralateral non-electroporated side. The asterisk points to dorsal dI3 Islet1/2^+^ post-mitotic neurons derived from pd3 progenitors that show no differences upon Dyrk1B overexpression. **C**, **D** Dyrk1B overexpression results in decreased Olig2^+^ MN progenitors (arrowhead) by 25.12% ± 3.11 (p ≤ 0.001, n = 17 sections from 4 embryos), compared to the contralateral non-electroporated SC side in Dyrk1B/GFP embryos. **E**, **F** Similarly, Dyrk1B overexpression results in decreased number of HB9^+^ MNs (arrowhead) by 20.33% ± 1.67 (p ≤ 0.001, n = 17 sections from 4 embryos), and in (**G**,** H**), decreased number of Islet1/2^+^ MNs (arrowhead) by 16.25% ± 3.60 (p ≤ 0.01, n = 15 sections from 4 embryos), as well as in (**I**, **J**), decreased number of Chx10^+^ V2a interneurons (INs) (arrowhead) by 39.33% ± 3.85 (p ≤ 0.001, n = 20 sections from 4 embryos). No differences between the two sides were observed in control GFP-electroporated embryos in all cases. Cell numbers are normalized to total cell nuclei. Scale bars: 100 µm. TO-PRO-3: nuclei staining. Data are mean ± SEM, *p ≤ 0.05, **p ≤ 0.01, ***p ≤ 0.001; ns, non-significant (two-tailed Unequal variance t test). Spinal cord sections correspond to the brachial level

As Dyrk1B has been previously shown to have an anti-apoptotic role [20] in myogenesis [22] and cancer [29, 30, 32, 33], we investigated if it has a similar function in the developing chick SC. Apoptosis of spinal MNs occurs during SC development at discrete developmental stages, along the rostrocaudal axis of the SC [68–70]. At E4 chick neural tube apoptosis occurs physiologically in the MN domain at the brachial level where the final number of MNs is stabilized at E15 [69]. To address if Dyrk1B overexpression has an effect on MN apoptosis, we compared the expression of activated Caspase-3 in the electroporated versus the non-electroporated side of SC in experimental and control embryos. First, we observed that in both groups, apoptosis was restricted in the MN domain in line with previous findings. Unexpectedly though, the percentage of Casp3^+^/Islet1/2^+^ cells over total nuclei was increased by 102.74% ± 17.02 (***p ≤ 0.001, n = 12 sections from 4 embryos) in the electroporated side of Dyrk1B/GFP embryos, compared to the contralateral side, while no significant differences were observed between the two sides of the SC in GFP embryos (Fig. 3A,B). Our data indicate that Dyrk1B overexpression promotes apoptosis in the MN domain, unlike the previously noted anti-apoptotic role of Dyrk1B, suggesting that Dyrk1B function on cell survival and apoptosis is tissue- and/or context-dependent.

To further investigate the effect of Dyrk1B on the MN domain, we compared bilaterally the Olig2^+^ MN progenitors in Dyrk1B/GFP and control GFP embryos. All quantifications in this and following sections were made by counting marker ^+^ cells normalized over total nuclei and given as percentages. We estimated that the percentage of Olig2^+^ MN progenitors in the electroporated side of Dyrk1B/GFP embryos was reduced by 25.12% ± 3.11 (***p ≤ 0.001, n = 17 sections from 4 embryos) as compared to the non-electroporated side, while no significant differences were observed bilaterally in the SC of GFP embryos (Fig. 3C, D). In agreement, the percentage of MNs positive for the homeobox transcription factor Hb9, which is expressed in newborn and late postmitotic MNs [71] was reduced by 20.33% ± 1.67 (***p ≤ 0.001, n = 17 sections from 4 embryos) at the Dyrk1B/GFP electroporated side compared to the non-electroporated side, while no significant differences were observed between the two sides of control GFP embryos (Fig. 3E, F). Moreover, the percentage of Islet1/2^+^ postmitotic MNs was also reduced by 16. 25% ± 3.60 (**p ≤ 0.01, n = 15 sections from 4 embryos) at the Dyrk1B/GFP electroporated side compared to the non-electroporated side, whereas no such differences were observed in GFP embryos (Fig. 3G, H). Markedly, the effect of forced Dyrk1B expression on the reduction of MN progenitors and MNs was most pronounced when Dyrk1B/GFP electroporation reached the most ventral parts of the SC, as revealed by the loss of Olig2^+^, Hb9^+^ and Islet1/2^+^ cells (see arrowheads in Fig. 3C, E, G).

An additional ventral phenotype was associated with the number of V2a interneurons (INs) expressing the transcription factor Chx10. On the Dyrk1B/GFP electroporated side, the percentage of Chx10^+^ interneurons was reduced by 39.33% ± 3.85 (***p ≤ 0.001, n = 20 sections from 4 embryos) compared to the non-electroporated side, while no significant differences were observed in GFP embryos (Fig. 3I, J). Our data suggest that Dyrk1B overexpression promotes apoptosis in the MN domain and decreases the numbers of pMNs, MNs, and V2a interneurons.

### Dyrk1B overexpression affects the ventral patterning of embryonic chick spinal cord

**Fig. 4:**
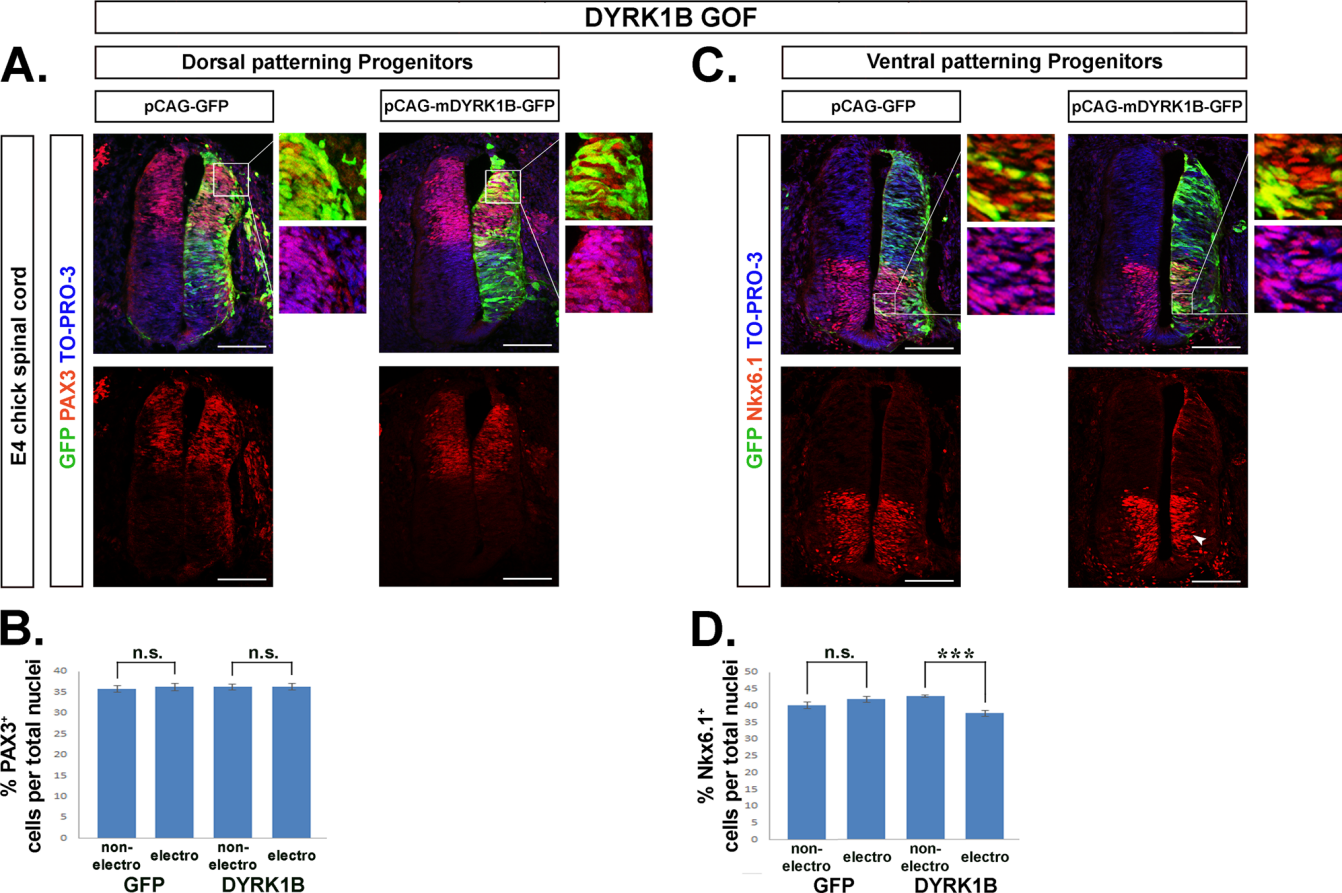
Dyrk1B overexpression affects the ventral patterning of embryonic chick spinal cord. **A**, **B** Dyrk1B overexpression does not affect dorsal patterning of spinal cord, as Pax3^+^ cells dorsally are not affected in the electroporated side of Dyrk1B/GFP embryos (p > 0.05, n = 20 sections from 4 embryos) when their number compared to the contralateral side. **C**, **D** In contrast, Dyrk1B overexpression reduces the number of Nkx6.1^+^ cells ventrally (arrowhead) by 12.18% ± 2.10 (p ≤ 0.001, n = 20 sections from 4 embryos) as compared to the non-electroporated contralateral SC side, while no differences are observed between the two sides in control GFP embryos. Cell numbers are normalized to total cell nuclei. Scale bars: 100 µm. TO-PRO-3: nuclei staining. Data are mean ± SEM, *p ≤ 0.05, **p ≤ 0.01, ***p ≤ 0.001; ns, non-significant (two-tailed Unequal variance t test). Spinal cord sections correspond to the brachial level

Next we addressed if Dyrk1B affects SC dorsoventral patterning by examining the expression of the paired-box transcription factor Pax3, which is expressed in dorsal progenitors, along with the expression of the homeodomain transcription factor Nkx6.1, which marks ventral progenitors and belongs to Class II transcription factors induced by Shh [71, 72]. We found that the percentage of Pax3^+^ cells was not affected in the Dyrk1B/GFP-electroporated side of E4 embryos when compared to the contralateral side (Fig. 4A, B). At the same time, the percentage of Nkx6.1^+^ cells was reduced by 12.18% ± 2.10 (***p ≤ 0.001, n = 20 sections from 4 embryos), (Fig. 4C, D), while no significant differences were observed bilaterally in control GFP embryos. To further verify that dorsal patterning is not affected by DyrK1B, we estimated the percentage of dI3 Islet1^+^post-mitotic neurons (indicated by an asterisk in Fig. 3A) derived from pd3 progenitors dorsally, and found that they were not different when compared to the contralateral side (Fig. S5). Taken together, our data indicate that Dyrk1B overexpression does not affect dorsal patterning while it influences ventral SC patterning.

### AZ191 increases the proliferation of ventral progenitors resulting in increased number of motor neurons

**Fig. 5:**
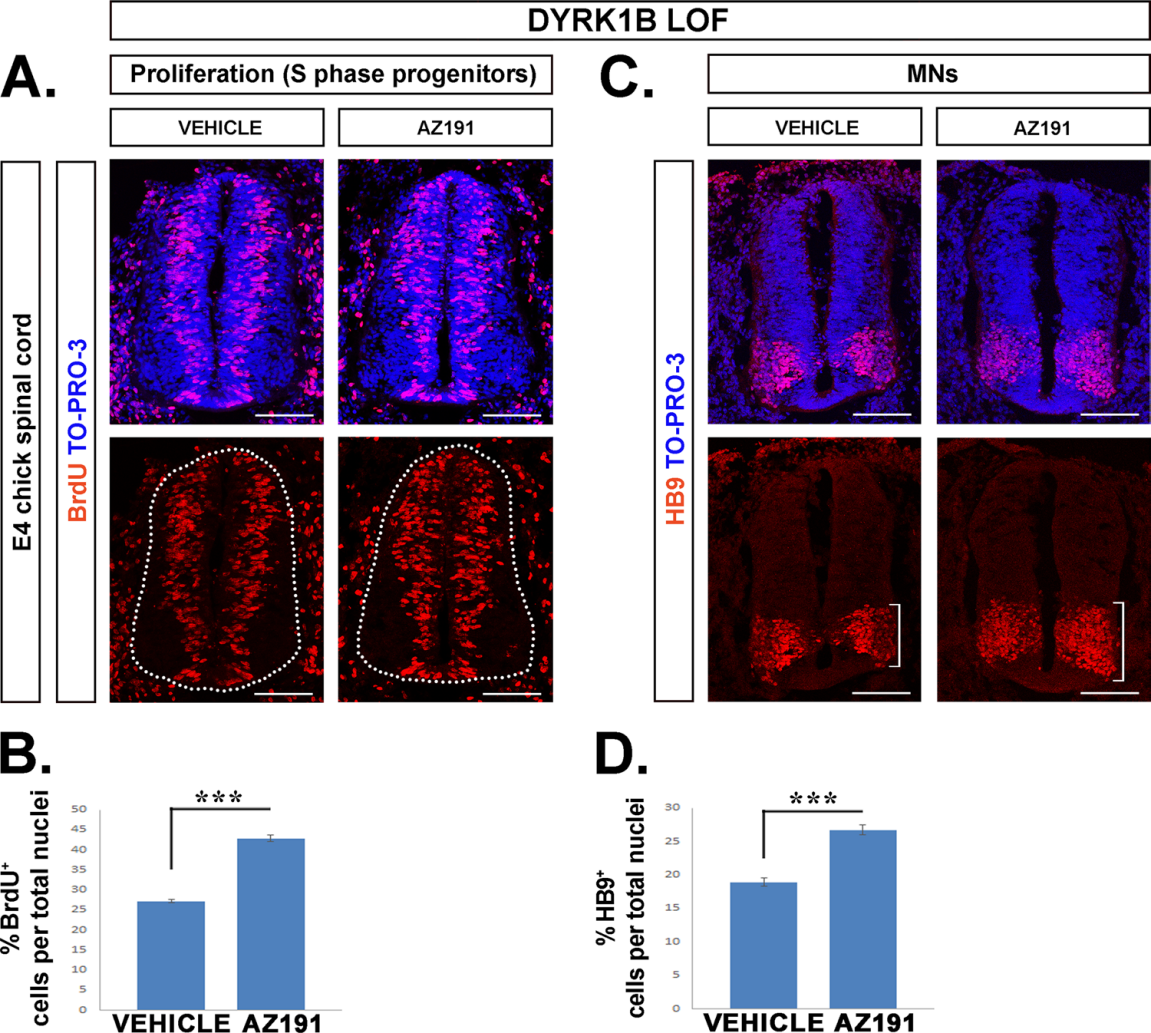
AZ191 increases the number of cycling progenitors resulting in increased motor neurons. **A**, **B** Inhibition of endogenous Dyrk1B kinase activity by administration of AZ191 inhibitor in the E2 spinal cord, results in increased number of BrdU^+^ cycling progenitors by 57.51% ± 3.05 (p ≤ 0.001, n = 22 sections from 4 embryos) as well as in **C**, **D** increased number of HB9^+^ post-mitotic MNs by 41.26% ± 4.07 (p ≤ 0.001, n = 17 sections from 4 embryos), when compared to control embryos treated with vehicle (DMSO) only. Cell numbers are normalized to total cell nuclei. Scale bars: 100 µm. TO-PRO-3: nuclei staining. Data are mean ± SEM, *p ≤ 0.05, **p ≤ 0.01, ***p ≤ 0.001; ns, non-significant (two-tailed Unequal variance t test). Spinal cord sections correspond to the brachial level

**Fig. 6:**
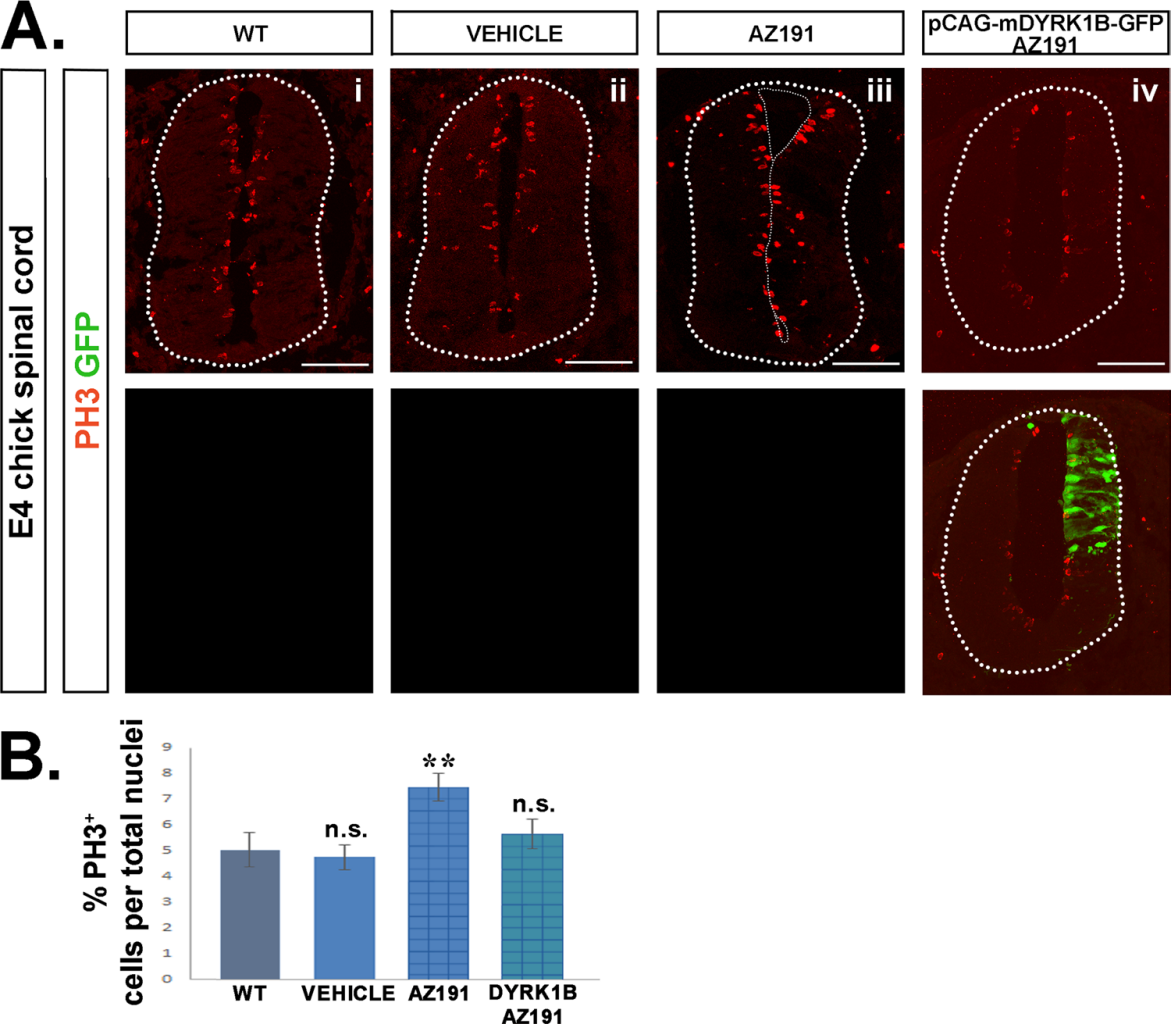
Dyrk1B electroporation reverses the phenotype of AZ191 inhibitor in cellular proliferation. **A** In *mechanistic phenotype rescue* experiments, AZ191 inhibitor was administered at E2 chick embryos 2 h after unilateral *in ovo* Dyrk1B/GFP electroporation, and the number of PH3^+^ mitotic progenitors located in the ventricular zone (VZ) was measured and compared to PH3^+^ progenitors in wild-type. Similarly, vehicle-only or AZ191-only treated embryos were analyzed and compared to wild-type. **B** Quantification shows that the increase in the number of PH3^+^ cells by 48.32% ± 10.69 (p ≤ 0.01, n = 12 sections from 4 embryos) observed in the presence of AZ191 (**A**iii), is reversed to wild-type levels (**A**i) by concurrent electroporation of Dyrk1B/GFP plasmid (**A**iv). Notably, PH3^+^ cells in DMSO-treated embryos (vehicle) (**A**ii) display non-significant differences as compared to wild-type (**A**i). Cell numbers are normalized to total cell nuclei. Scale bars: 100 µm. Data are mean ± SEM, *p ≤ 0.05, **p ≤ 0.01, ***p ≤ 0.001; ns, non-significant (two-tailed Unequal variance t-test). Spinal cord sections correspond to the brachial level

**Fig. 7:**
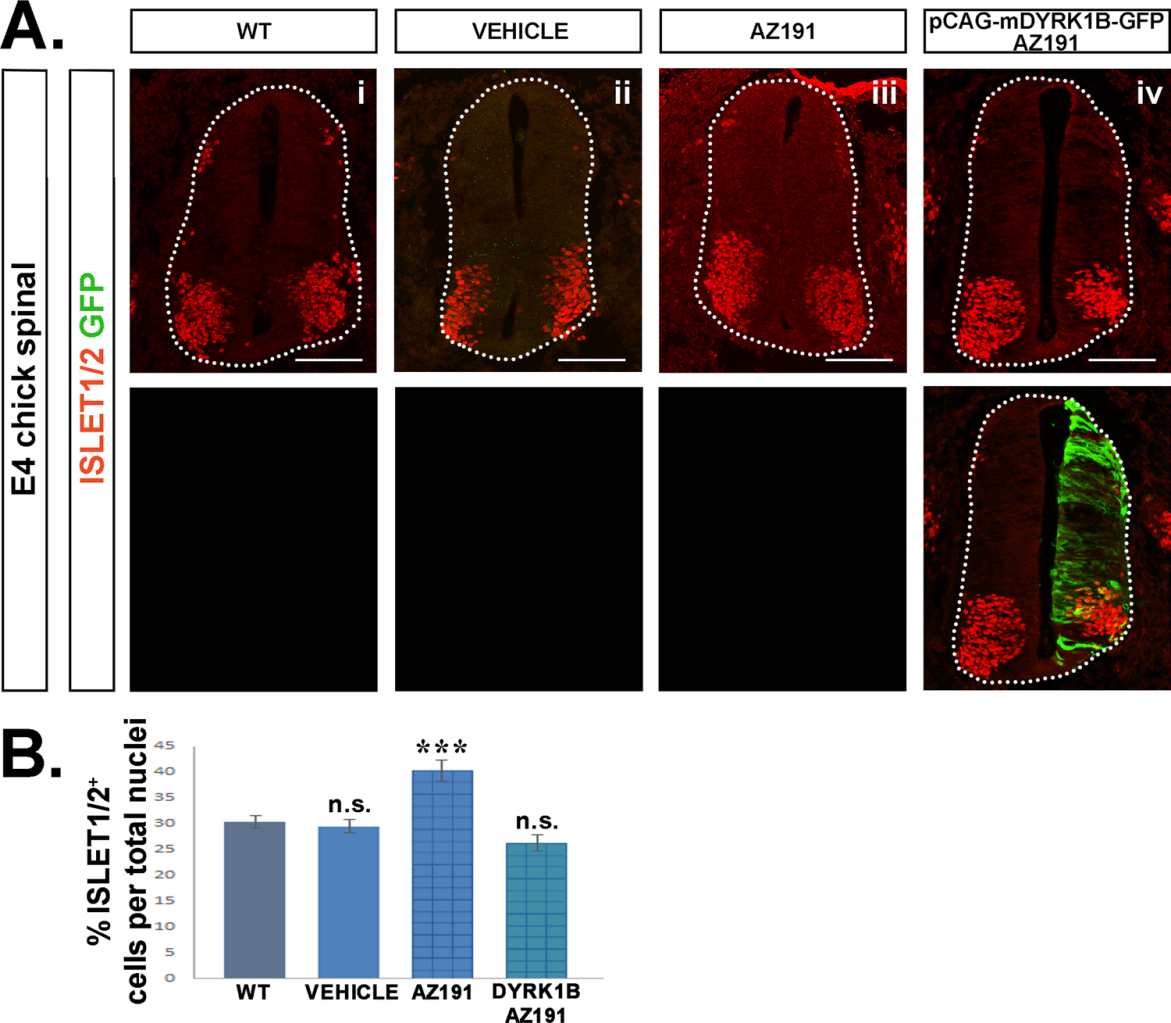
Dyrk1B overexpression reverses the phenotype of AZ191 in motor neurons. **A** In mechanistic phenotype rescue experiments, AZ191 inhibitor was administered at E2 chick embryos 2 h after unilateral *in ovo* Dyrk1B/GFP electroporation, and the number of Islet1/2^+^ MNs was measured and compared to Islet1/2^+^ MNs in wild-type. **B** The increased number of Islet1/2^+^ MNs by 33.12 ± 6.86 (p ≤ 0.001, n = 14 sections from 4 embryos), observed in the presence of AZ191 (**A**iii), was completely rescued by Dyrk1B electroporation (**A**iv). Notably, Islet1/2^+^ MNs in DMSO-treated embryos (vehicle) (**A**ii) display non-significant differences as compared to wild-type (**A**i). Cell numbers are normalized to total cell nuclei. Scale bars: 100 µm. Data are mean ± SEM, *p ≤ 0.05, **p ≤ 0.01, ***p ≤ 0.001; ns, non-significant (two-tailed Unequal variance t-test). Spinal cord sections correspond to the brachial level

To confirm Dyrk1B function in SC development, we performed *loss-of-function experiments* by applying pharmacological inhibition of the endogenous Dyrk1B kinase activity using AZ191, a potent small molecule inhibitor that selectively inhibits Dyrk1B [73, 74]. Chick embryos were injected with AZ191 at E2 and 48 h later the effect of pharmacological inhibition of endogenous Dyrk1B was analyzed by comparing AZ191-treated to control DMSO-treated embryos (vehicle). First, we investigated the effect of AZ191 on cellular proliferation by assessing the total number of BrdU^+^ cells following a 2-h BrdU pulse. The percentage of BrdU^+^ cells in AZ191-treated embryos was increased by 57.51% ± 3.05 (***p ≤ 0.001, n = 22 sections from 4 embryos), compared to DMSO-treated embryos (Fig. 5A, B). In accordance, the percentage of PH3^+^ cells was also increased by 48.32 ± 10.69 (**p ≤ 0.01, n = 12 sections from 4 embryos) (Fig. 6A, B). Notably, no differences were observed in BrdU^+^ (data not shown) and PH3^+^ cells (Fig. 6A, B), between DMSO-treated and wild-type (wt) chick embryos. Next, we investigated the effect of AZ191 on ventral progenitors p2, pMN and p3. In AZ191-treated embryos the percentages of p2 (Nkx6.1^+^/Olig2^−^), pMN (Nkx6.1^+^/Olig2^+^) and p3 (Nkx6.1^+^/Olig2^−^) progenitors were increased by 32.57% ± 9.86% (**p ≤ 0.01, n = 23 sections from 4 embryos), 31.90% ± 4.89 (***p ≤ 0.001, n = 23 sections from 4 embryos) and by 61.25 ± 23.75 (*p ≤ 0.05, n = 23 sections from 4 embryos) respectively, compared to DMSO-treated embryos (Fig. S6). Furthermore, we investigated the effect of AZ191 on the MN domain. In AZ191-treated embryos the percentage of Islet1/2^+^ MNs over total nuclei was increased by 33.12% ± 6.86 (***p ≤ 0.001, n = 14 sections from 4 embryos) (Fig. 7A, B) and the percentage of Hb9^+^ early and late MNs was increased by 41.26% ± 4.07 (***p ≤ 0.001, n = 17 sections from 4 embryos) (Fig. 5C, D), when compared to DMSO-treated embryos. In all cases DMSO-treated embryos showed no differences when compared with wt embryos. Our data suggest that inhibition of endogenous Dyrk1B increases the proliferation of neuronal progenitors, which in the ventral spinal cord results in increased numbers of p2, pMN, p3 progenitors and MNs (Hb9^+^ and Islet1/2^+^ cells).

### Dyrk1B overexpression reverses the phenotype of AZ191 in the embryonic chick spinal cord

To confirm the specificity of AZ191 inhibitor for Dyrk1B kinase activity, we performed *mechanistic phenotype rescue* experiments. To this end, E2 chick embryos were subjected to *unilateral in ovo* Dyrk1B/GFP electroporation followed 2 h later by AZ191 injection, and were compared to embryos treated with DMSO-vehicle or AZ191 only*.* Notably, the increase in PH3^+^ cells observed in the presence of AZ191, was rescued by concurrent electroporation of Dyrk1B/GFP (Fig. 6). In particular, no significant differences in PH3^+^ cells were observed between wild-type or vehicle-treated embryos, while in AZ191-treated embryos the percentage of PH3^+^ cells was increased by 48.32 ± 10.69 (**p ≤ 0.01, n = 12 sections from 4 embryos), as previously discussed. This effect was restored by Dyrk1B/GFP electroporation (Fig. 6B). Similarly, the AZ191-mediated increase in the percentage of Islet1/2^+^ MNs by 33.12% ± 6.86 (***p ≤ 0.001, n = 14 sections from 4 embryos), was also restored upon Dyrk1B/GFP electroporation (Fig. 7B). Notably, no significant differences were observed in the percentage of Islet1/2^+^ post-mitotic MNs between wild-type or vehicle-treated embryos.

### Dyrk1B kinase is a suppressor of Sonic Hedgehog signaling in the embryonic chick spinal cord

Shh produced initially by the notochord (NC) and later by medial (MFP) and lateral floor plate (LFP), forms a gradient [3, 6–10] that patterns the ventral neural tube into distinct progenitor domains, by controlling the expression of specific homeodomain and basic helix-loop-helix (bHLH) transcriptions factors [2, 3, 5, 6, 71, 72].

**Fig. 8:**
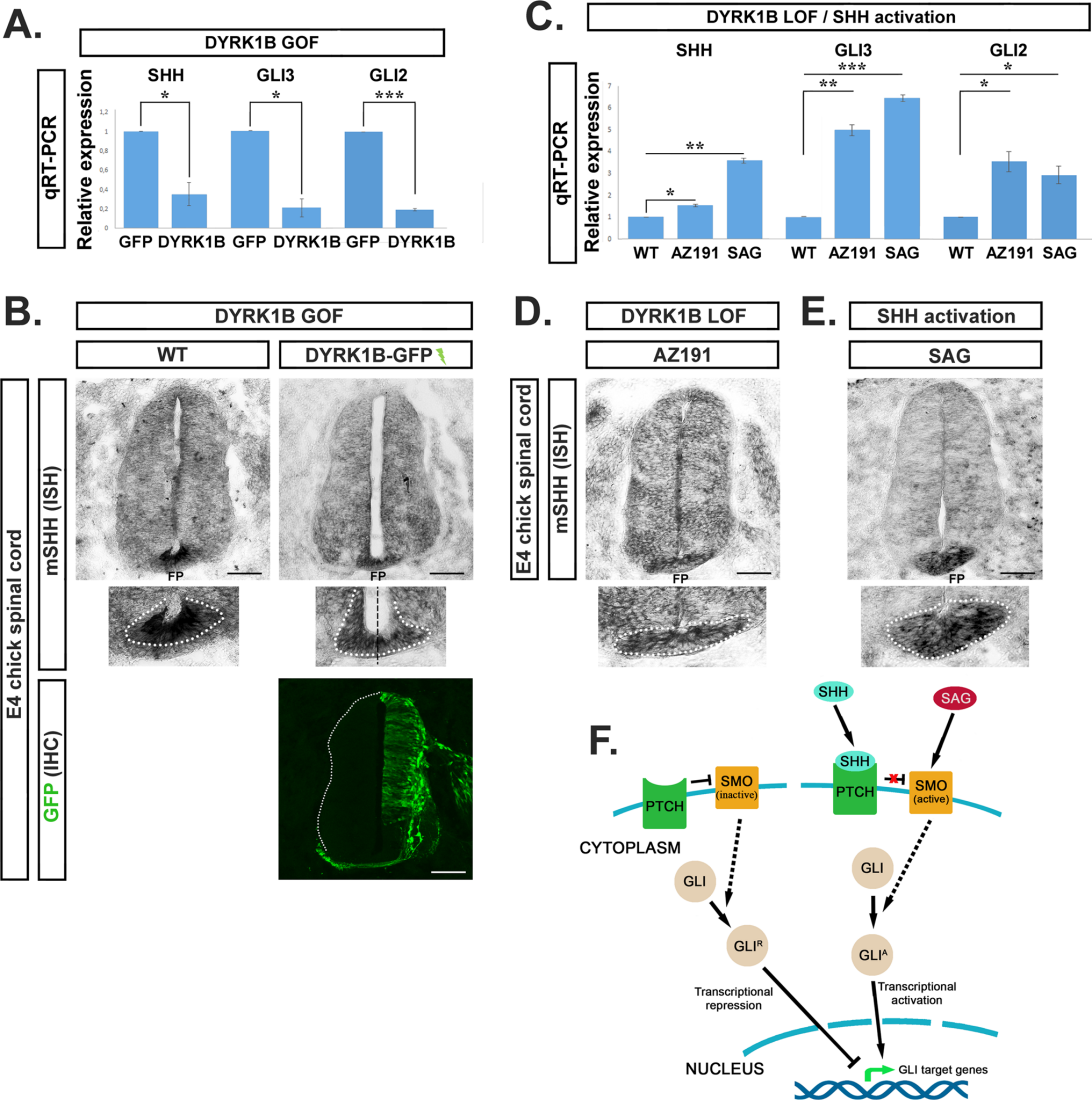
Dyrk1B acts as transcriptional suppressor of Shh, Gli2 and Gli3 in the embryonic chick spinal cord.** A** Dyrk1B overexpression (GOF) at E2 reduced at E4 Shh, Gli2 and Gli3 mRNA expression levels by 2.86-fold ± 0.99 (p ≤ 0.05, n = 3 independent experiments), by 5.28-fold ± 0.31 (p ≤ 0.001, n = 3 independent experiments), and by 4.76-fold ± 0.61 (p ≤ 0.05, n = 3 independent experiments) respectively, relatively to control GFP embryos, as determined by qRT-PCR analysis **B** In agreement, in situ hybridization (ISH) shows that Shh mRNA expression is decreased at the floor plate (FP) of the electroporated side in Dyrk1B/GFP embryos compared to the non-electroporated side, as well as to wild-type embryos. **C** Conversely, inhibition of endogenous Dyrk1B kinase activity by AZ191 (LOF) results in increased Shh, Gli2 and Gli3 mRNA expression levels by 1.52-fold ± 0.06 (p ≤ 0.05, n = 3 independent experiments), by 3.54-fold ± 0.47 (p ≤ 0.05, n = 3 independent experiments) and 4.85-fold ± 0.25 (p ≤ 0.01, n = 3 independent experiments) respectively, relatively to wild-type, as determined by qRT-PCR analysis. Similarly, activation of Shh pathway at the level of Smoothened (SMO), by the agonist SAG increased Shh, Gli2 and Gli3 mRNA expression levels in SAG-treated embryos by 3.54-fold ± 0.11 (p ≤ 0.01, n = 3 independent experiments), by 2.92-fold ± 0.41 (p ≤ 0.05, n = 3 independent experiments) and 6.30-fold ± 0.12 (p ≤ 0.001, n = 3 independent experiments) respectively, compared to wild-type, as revealed by qRT-PCR analysis. **D**, **E** In agreement, Shh mRNA expression levels were increased at the FP in AZ191 and SAG-treated embryos compared to wild-type, as revealed by ISH. Scale bars: 100 µm. Data are mean ± SEM, *p ≤ 0.05, **p ≤ 0.01, ***p ≤ 0.001; ns, non-significant (two-tailed Unequal variance t-test). Spinal cord sections correspond to the brachial level. **F** Schematic representation of canonical Shh signaling pathway. SMO is activated upon SHH binding to PTCH or directly by SAG, resulting in transcriptional activation of GLI target genes

A close regulatory link between Dyrk1B kinase and Shh/Gli signaling has been previously demonstrated in cancer [44, 50–53]. Thus, we speculated the involvement of Dyrk1B in Shh/Gli signaling given that Dyrk1B overexpression has a pronounced effect in ventral SC. To test our hypothesis, we performed real-time qRT-PCR analysis to estimate Shh, Gli2 and Gli3 mRNA levels upon forced expression of Dyrk1B and observed a significant downregulation of Shh mRNA expression levels by 2.86-fold ± 0.99 (*p ≤ 0.05, n = 3 independent experiments), of Gli2 mRNA levels by 5.28-fold ± 0.31 (*** p ≤ 0.001, n = 3 independent experiments) and of Gli3 mRNA levels by 4.76-fold ± 0.61 (*p ≤ 0.05, n = 3 independent experiments), as compared to GFP embryos (Fig. 8A). Furthermore, we performed ISH for detecting Shh mRNA expression in wild-type and Dyrk1B/GFP embryos. In agreement with the real-time qRT-PCR results, the expression of Shh mRNA in the FP was reduced at the Dyrk1B/GFP-electroporated side when compared with the contralateral side and with wild-type SC (Fig. 8B). On the other hand, in AZ191-treated embryos, Shh, Gli2 and Gli3 mRNA levels were increased by 1.52-fold ± 0.06 (*p ≤ 0.05, n = 3 independent experiments), by 3.54-fold ± 0.47 (*p ≤ 0.05, n = 3 independent experiments) and by 4.85-fold ± 0.25 (**p ≤ 0.01, n = 3 independent experiments) respectively, when compared to wild-type (Fig. 8C), as estimated by qRT-PCR. The increase in Shh mRNA levels was furthermore confirmed by ISH (Fig. 8D). This data suggests interception of Dyrk1B and Shh/Gli signaling pathways.

### Administration of SAG rescues the ventral phenotype of Dyrk1B overexpression

**Fig. 9:**
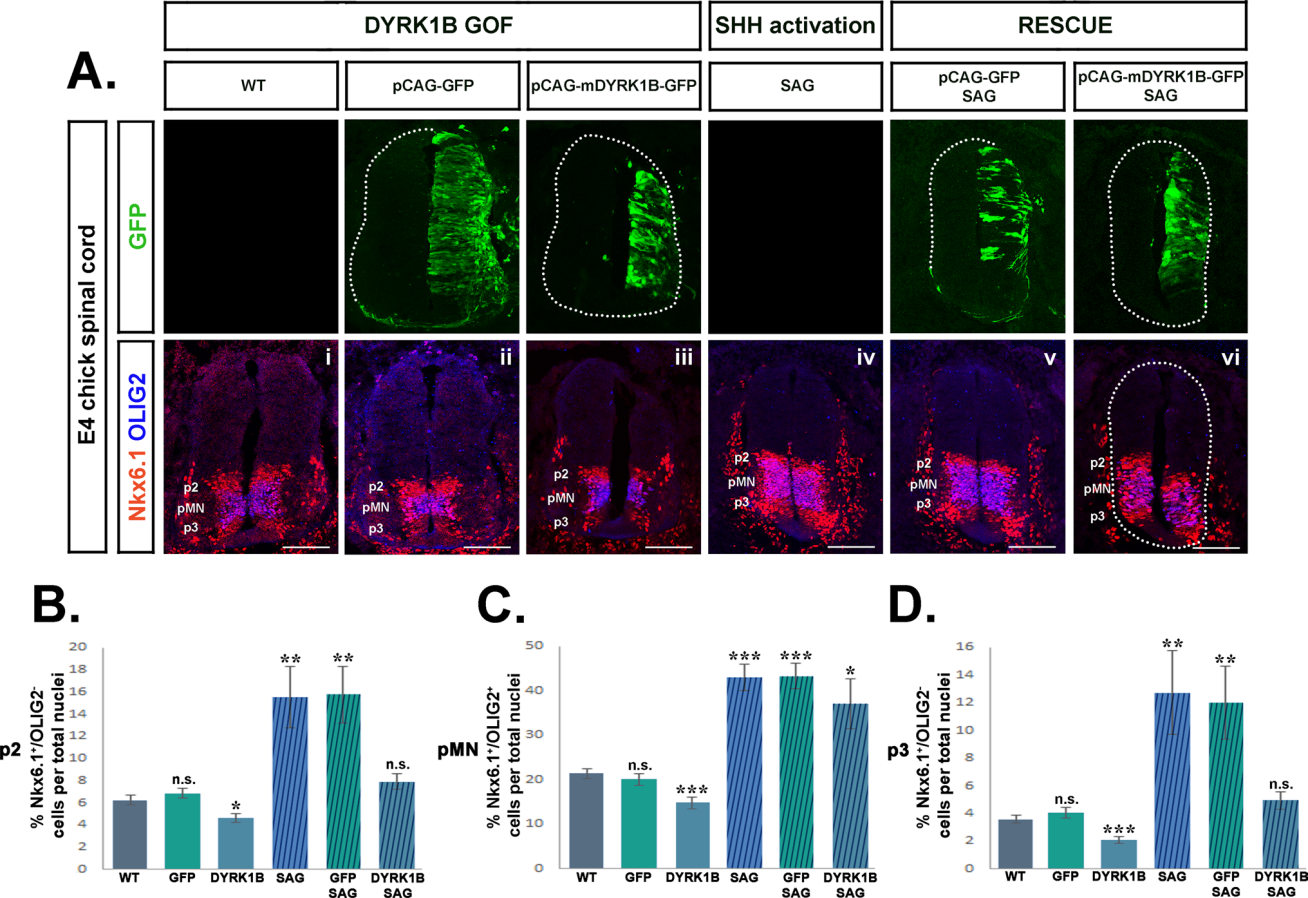
SAG rescues the ventral phenotype of Dyrk1B overexpression. **A** Effect of SAG administration alone or after concurrent electroporation with either Dyrk1B/GFP or GFP expression plasmids, on the number of p2, pMN and p3 ventral progenitors at E4. Upper panel: GPF immunofluorescence shows the extent of electroporation in each case. Lower panel: p2, pMN and p3 domains are distinguished by double immunofluorescence for Nkx6.1 and Olig2 transcription factors, as indicated. Nkx6.1 is expressed by p2, pMN and p3, while Olig2 marks exclusively the pMN domain. **B** Dyrk1B overexpression (GOF) results in reduced p2 progenitors (Nkx6.1^+^/Olig2^−^ upper cell population) by 25.88% ± 6.87 (p ≤ 0.05, n = 15 sections from 4 embryos) (**A**iii), while SAG administration results in increased p2 progenitors by 148.24% ± 43.93 (p ≤ 0.01, n = 15 sections from 4 embryos) (**A**iv), both compared to wild-type (**A**i). SAG administration (**A**vi) in Dyrk1B/GFP embryos restored completely p2 progenitors at normal levels. Notably, in GFP embryos the p2 population (**A**ii) was similar to wild-type (**Ai**), while in GFP embryos that were subsequently treated with SAG **(Av)** p2 progenitors were increased by 151.76% ± 40.89 (p ≤ 0.01, n = 15 sections from 4 embryos). **C** Dyrk1B overexpression (GOF) resulted in reduced pMN progenitors (Olig2^+^ cell population) by 30.47% ± 6.07 (p ≤ 0.001 n = 15 sections from 4 embryos) (**A**iii), and SAG administration resulted in increased pMN progenitors by 100.93% ± 13.46 (p ≤ 0.001, n = 15 sections from 4 embryos) (**A**iv), both compared to wild-type (**A**i). SAG administration in Dyrk1B/GFP embryos **(Avi)** resulted in increased pMN progenitors by 73.79% ± 26.17 (p ≤ 0.05, n = 15 sections from 4 embryos) compared to wild-type (**A**i). Notably, in GFP embryos the pMN population (**A**ii) was similar to wild-type (**Ai**), while in GFP embryos that were subsequently treated SAG (**A**v) pMN progenitors were increased by 102.20% ± 13.50 (p ≤ 0.001, n = 15 sections from 4 embryos). **D** Dyrk1B overexpression (GOF) resulted in reduced p3 progenitors (Nkx6.1^+^/Olig2^−^ lower cell population) by 41.39% ± 7.50 (p ≤ 0.001, n = 15 sections from 4 embryos) (**A**iii), SAG administration resulted in increased p3 progenitors by 253.89% ± 84.17 (p ≤ 0.01, n = 15 sections from 4 embryos) (**A**iv), both compared to wild-type (**A**i). SAG administration (**A**vi) in Dyrk1B/GFP embryos resulted in non-significant differences in the p3 population compared to wild-type (**A**i). Notably, in GFP embryos the p3 population (**A**ii) was similar to wild-type (**A**i), while in GFP embryos that were subsequently treated with SAG (**A**vi), p3 progenitors were increased by 232.78% ± 73.33 (p ≤ 0.01, n = 15 sections from 4 embryos). Cell numbers are normalized to total cell nuclei. Scale bars: 100 µm. Data are mean ± SEM, *p ≤ 0.05, **p ≤ 0.01, ***p ≤ 0.001; ns, non-significant (two-tailed Unequal variance t test). Spinal cord sections correspond to the brachial level

To further investigate the interference of Dyrk1B in Shh/Gli signaling, we proceeded to activate the Shh pathway at the level of Smoothened, which induces downstream Gli transcriptional effectors (Fig. 8F). For this purpose, 1 µg of Smoothened agonist (SAG) was administered at E2 embryos, and its effect was examined on Shh signaling at E4. We observed a transcriptional positive feedback loop in Shh, Gli2 and Gli3 mRNA expression, induced by Smoothened activation with SAG, which resulted in increased Shh, Gli2 and Gli3 mRNA levels by 3.54-fold ± 0.11 (**p ≤ 0.01, n = 3 independent experiments), by 2.92-fold ± 0.41 (*p ≤ 0.05, n = 3 independent experiments) and by 6.30-fold ± 0.12 (***p ≤ 0.001, n = 3 independent experiments), respectively, as determined by qRT-PCR (Fig. 8C). Moreover, by performing ISH, we confirmed the increased Shh mRNA expression in the FP of SAG-treated embryos (Fig. 8E). As before with mRNA levels, we also observed a similar trend by Western blot, showing that Dyrk1B overexpression or loss of function, as well as SAG treatment, affect the ratio of activator to repressor Gli3 (Gli3A/Gli3R) protein forms and the expression of the early responding proximal targets of Shh signaling, FoxA2 and Nkx2.2, while in *phenotype rescue* experiments these proteins seem to be restored to control levels (Fig.S7). Next we tested whether activation of SMO by SAG could rescue the phenotype in Dyrk1B/GFP embryos. For this purpose, 1 µg SAG was administered at E2 chick embryos 2 h after unilateral *in ovo* electroporation with Dyrk1B/GFP or GFP expression plasmids. Then, we analyzed at E4 the effect of SAG administration either alone or together with Dyrk1B/GFP electroporation. GFP and wild-type embryos served as additional controls. Since Shh is a master regulator of ventral SC patterning, we examined the number of p3, pMN and p2 progenitors characterizing distinct ventral domains (Fig. 9A). We distinguished their boundaries using double immunostaining for Nkx6.1 and Olig2 transcription factors. Nkx6.1 is expressed by p3, pMN and p2 domains, while Olig2 expression marks only the pMN domain (Fig. 9Ai–vi). In all cases examined, we attested that SAG reversed the phenotype induced by forced Dyrk1B expression.

In particular, the percentage of p2 progenitors over total nuclei was decreased by 25.88% ± 6.87 (*p ≤ 0.05, n = 15 sections from 4 embryos) in Dyrk1B/GFP embryos and, conversely, was increased by 148.24% ± 43.93 (**p ≤ 0.01, n = 15 sections from 4 embryos) in SAG only-treated embryos, compared to wild-type embryos (Fig. 9B). In *phenotype rescue* experiments, in which Dyrk1B/GFP electroporation was followed by SAG, SAG was sufficient to restore the p2 population to wt levels (Fig. 9B). Notably, in control GFP embryos the p2 progenitor population was as in wt, while in GFP embryos that were treated with SAG, the percentage of p2 progenitors was increased by 151.76% ± 40.89 (**p ≤ 0.01, n = 15 sections from 4 embryos), similarly to wt (Fig. 9B).

Likewise, the percentage of p3 progenitors that was decreased by 41.39% ± 7.50 (***p ≤ 0.001, n = 15 sections from 4 embryos) in Dyrk1B/GFP embryos, was restored at wt levels after administration of SAG (Fig. 9D). At the same time, in SAG only-treated embryos p3 progenitors were increased dramatically by 253.89% ± 84.17 (**p ≤ 0.01, n = 15 sections from 4 embryos) (Fig. 9D). Additionally, in control GFP embryos, the p3 population was similar to wild-type, while in GFP embryos that were treated with SAG, the percentage of p3 progenitors was increased by 232.78 ± 73.33 (**p ≤ 0.01, n = 15 sections from 4 embryos) (Fig. 9D).

Finally, the percentage of pMN progenitors that was decreased by 30.47% ± 6.07 (***p ≤ 0.001, n = 15 sections from 4 embryos) in Dyrk1B/GFP embryos, was increased by SAG treatment even further than wt levels, by 73.79% ± 26.17 relatively to wt (*p ≤ 0.05, n = 15 sections from 4 embryos). In addition, the percentage of pMN progenitors was increased by 100.93% ± 13.46 (***p ≤ 0.001, n = 15 sections from 4 embryos) relatively to wt in SAG only-treated embryos (Fig. 9C). Notably, in control GFP embryos the pMN population was similar to wild-type, while in GFP embryos treated with SAG, the normalized number of pMN progenitors was increased to similar levels as with SAG alone, by 102.20% ± 13.50 (***p ≤ 0.001, n = 15 sections from 4 embryos) (Fig. 9C). Thus, our data demonstrate that activation of the Shh pathway restores the phenotype of Dyrk1B overexpression.

### Dyrk1B affects the size of LMCm via Sonic hedgehog signaling

**Fig. 10:**
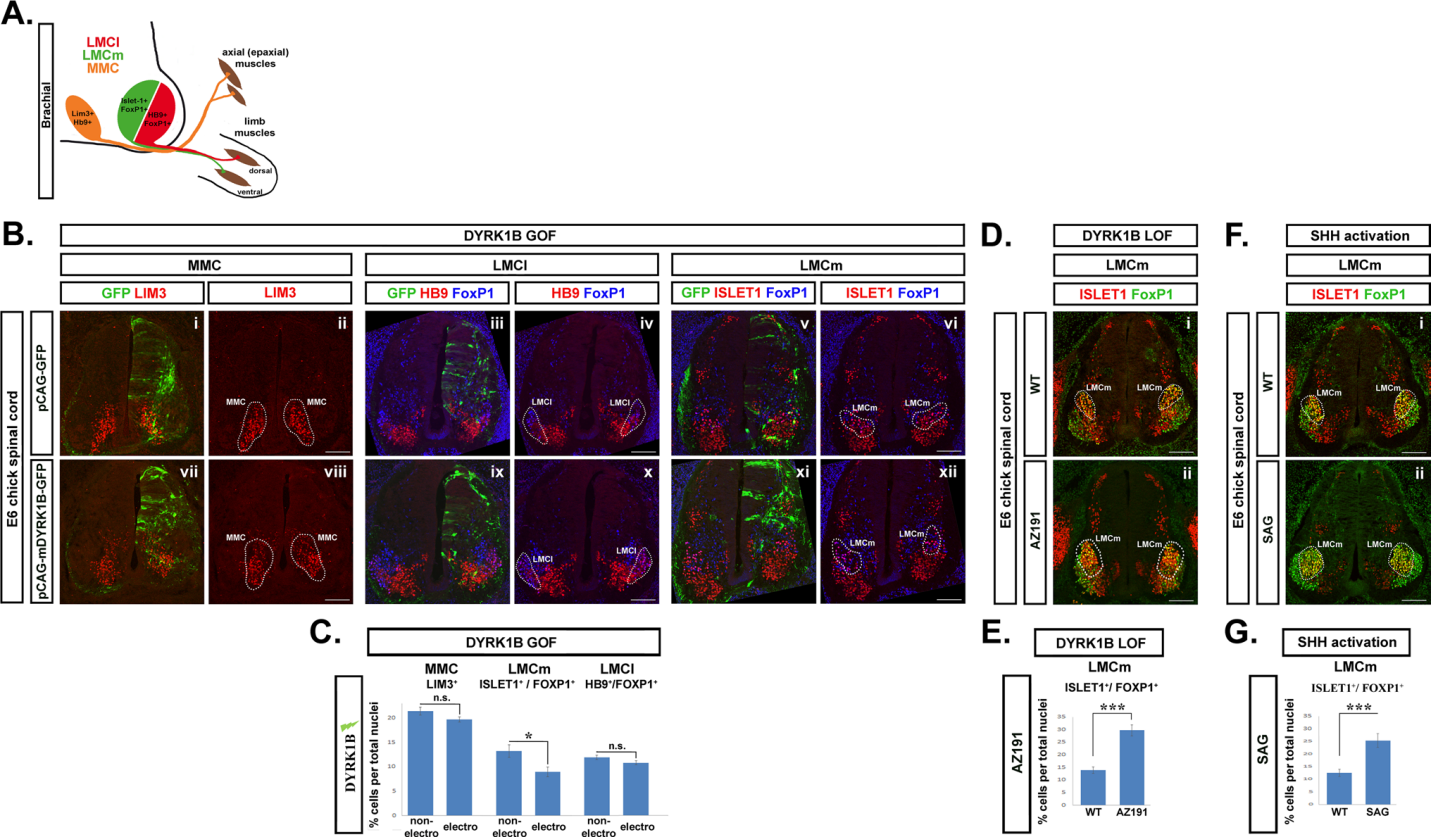
Dyrk1B affects the number of LMCm motor neurons at E6 chick spinal cord. **A** Anatomical map of motor columns and their peripheral targets at the brachial level of E6 chick spinal cord. **B**, **C** Dyrk1B overexpression (GOF) at E2 reduced specifically at E6 the number of LMCm MNs (Foxp1^+^/Islet1^+^) (**A**xi, xii), 32.05% ± 7.48 (p ≤ 0.05, n = 23 sections from 4 embryos), while had no effect on LMCl (HB9^+^/Foxp1^+^) (**A**ix, x) and MMC (Lim3^+^) (**A**vii, viii) MNs. Comparisons were made between the electroporated vs the non-electroporated side in each case. In control GFP embryos no significant differences were observed in LMCm, LMCl and MMC MNs (**A**i–vi) between the two sides of SC. **D**,** E** AZ191 administration at E2 (LOF), results in the opposite phenotype at E6, increasing specifically the number of LMCm MNs (Foxp1^+^/Islet1^+^) (**D**i–ii) by 113.49% ± 15.49 (p ≤ 0.001, n = 19 sections from 4 embryos) compared to wild-type. **F**,** G** Similarly, SAG administration at E2, results at E6 in increased number of LMCm MNs (**F**i–ii) by 101.43% ± 22.04 (p ≤ 0.001, n = 19 sections from 4 embryos) compared to wild-type. Notably, AZ191 and SAG administration had no effect on MMC and LMCl MNs (see Fig. S8). Cell numbers are normalized to total cell nuclei. Scale bars: 100 µm. Data are mean ± SEM, *p ≤ 0.05, **p ≤ 0.01, ***p ≤ 0.001; ns, non-significant (two-tailed Unequal variance t test). Spinal cord sections correspond to the brachial level

Spinal MNs are organized into four motor columns extending along the rostrocaudal axis of SC, the median (MMC), lateral (LMC), hypaxial (HMC), and preganglionic motor columns (PGC). LMC MNs subdivide at the brachial and lumbar levels of SC into medial LMC (LMCm) and lateral LMC (LMCl) columns that innervate the muscle limbs ventrally and dorsally, respectively [70, 75–80] (Fig. 10A). Having demonstrated that Dyrk1B overexpression promotes the reduction of Olig2^+^ pMNs as well as of Hb9^+^ and Islet1/2^+^ MNs, we asked whether the forced expression of Dyrk1B has an effect on the columnar organization of MNs at a later developmental stage when motor columns have been formed. To this end, we performed immunostainings at E6 SC cryosections corresponding to the brachial level, for transcription factors characterizing each motor column. Surprisingly, we observed that the loss of MNs at E4, was selectively reflected in LMCm (Foxp1^+^/Islet1^+^) MNs, which were reduced by 32.05% ± 7.48% (*p ≤ 0.05, n = 23 sections from 4 embryos), (Fig. 10Bvi, xii, Fig. 10C). In contrast, LMCl (Foxp1^+^/Hb9^+^) MNs (Fig. 10Biv, x, C) and MMC (Lim3^+^/Hb9^+^) MNs (Fig. 10Bii, viii,C) were not affected by Dyrk1B overexpression (Fig. 10C). All cell numbers are normalized to total cell nuclei.

Conversely, AZ191 administration resulted in the opposite phenotype with increased percentage of LMCm MNs by 113.49% ± 15.49 (***p ≤ 0.001, n = 19 sections from 4 embryos) (Fig. 10D, E), compared to wild-type embryos. Interestingly, activation of the Shh pathway using SAG, phenocopied the effect of AZ191 and increased the percentage of LMCm MNs by 101.43% ± 22.04 (***p ≤ 0.001, n = 19 sections from 4 embryos) (Fig. 10F, G). Neither AZ191 (Fig. S8A, S8B) nor SAG (Fig.S8C, S8D) had any effect on LMCl and MMC MNs. Together, our data suggest that Dyrk1B is critical for the control of ventral progenitors by regulating Shh, Gli2 and Gli3 transcriptional levels thereby affecting the generation of MNs, and appears also vital for LMCm MN subtype specification, presumably via the same pathway.

**Fig. 11:**
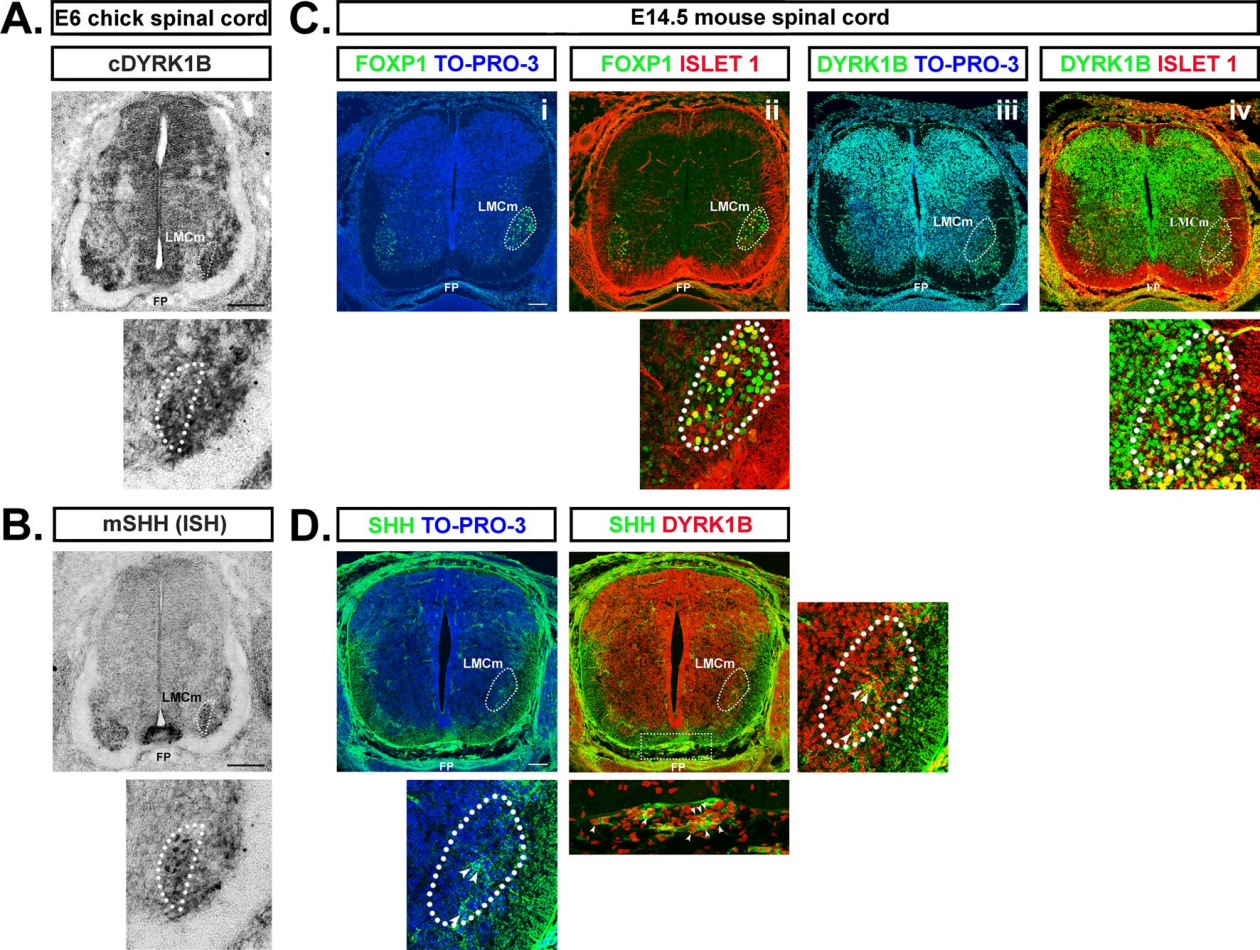
Sonic Hedgehog and Dyrk1B are co-expressed by LMCm motor neurons and floor plate in the embryonic spinal cord. **A**, **B** At E6 chick spinal cord Dyrk1B mRNA is expressed in the whole MN domain including LMCm MNs (depicted by white dots), while Shh mRNA expression is restricted in LMCm MNs (depicted by white dots), as revealed by in situ hybridization (ISH) on transverse spinal cord brachial sections. Intense signal was also observed for Dyrk1B and Shh mRNA in the floor plate. ISH for Dyrk1B and Shh mRNAs was performed using a chick Dyrk1B and a mouse Shh riboprobe, respectively. **C** At E14.5 mouse spinal cord (corresponds to E6 in chick), Dyrk1B is expressed by LMCm MNs (Foxp1^+^/Islet1^+^), as revealed by immunohistochemistry (IHC). **D** At E14.5 mouse spinal cord Shh and Dyrk1B are co-expressed by cells of the floor plate and LMCm region (indicated by arrowheads in the magnification). Scale bars: 100 µm. Spinal cord sections correspond to the brachial level

Indeed, Shh is expressed by MNs at brachial and lumbar levels when motor columnar identities are established and is required for proper LMC formation [81]. Therefore, we investigated Dyrk1B and Shh mRNA expression by ISH in transverse cryosections from the brachial level of E6 chick and E14.5 mouse SC. This showed that Shh mRNA is co-expressed with Dyrk1B in FP and LMCm MNs (Fig. 11). Thus, the selective effect of Dyrk1B in LMCm motor column, could be explained by its repressive action on endogenous Shh expression in LMCm MNs.

## Discussion

In this study we revealed a novel role for Mirk/Dyrk1B kinase in SC development, by applying *in ovo gain-and-loss-of-function* and *phenotype rescue* experiments in the chick embryo. We found that Dyrk1B is expressed by NC, FP, proliferating progenitors in VZ and by post-mitotic neurons, including MNs. We showed that ectopic overexpression of Dyrk1B promotes cell cycle exit and neuronal differentiation. These observations are in accordance with Dyrk1B main known function, as a negative cell cycle regulator [16, 20, 44, 45] during myogenesis [21–23], spermatogenesis [24], in mouse neuroblastoma cells [42] and cancer [29–31, 38]. Dyrk1B acts on the cell cycle by phosphorylating cyclin D1 [40, 42–45] and p27^Kip1^ [39], leading to their degradation and stability, respectively, and by activating together with Dyrk1A, the DREAM complex, which is essential for maintaining the quiescent state [82–84]. Our data in this study are also in accordance with our previous findings, concerning Dyrk1B function in neural cells [42–44]. Specifically, we have demonstrated that Dyrk1B overexpression in mouse neuroblastoma cells promotes cell cycle exit and neuronal differentiation by phosphorylating cyclin D1, followed by its cytoplasmic relocation and subsequent degradation by the 26S proteasome. This negative effect on cell cycle progression is reversed by Dyrk1B interacting partner, the scaffolding protein RanBPM [42].

Of interest, our present study reveals novel findings indicating that Dyrk1B overexpression in embryonic chick SC promotes apoptosis specifically in the MN domain. Increased apoptosis in the MN domain combined with premature cell cycle exit, resulted in decreased number of p2, pMN and p3 ventral progenitors and consequently reduced post-mitotic INs and MNs, respectively. Conversely, inhibition of endogenous Dyrk1B activity, by AZ191 selective inhibitor, resulted in increased proliferation and increased p2, pMN, and p3 progenitors as well as increased Hb9^+^ and Islet1/2^+^ MNs and at a later stage increased LMCm MNs. Although previous studies in myogenesis [22] and cancer [29, 30, 32, 33] have reported an anti-apoptotic role for Dyrk1B, here we observed that Dyrk1B overexpression increases the naturally occurring apoptosis during SC development in the MN domain, suggesting that Dyrk1B function in cell survival is tissue-and/or context-dependent. In addition, Dyrk1B-induced apoptosis in the MN domain, as well as its powerful ventral phenotype, indicated that it may be related to compromised Shh signaling, as Shh is crucial for cell survival of ventral progenitors and MNs [7–11]. Moreover, we have demonstrated that Dyrk1B overexpression decreases the number of Nkx6.1^+^ cells ventrally, while it does not affect the number of Pax3^+^ progenitor cells, or the number of dI3 Islet1^+^ neurons dorsally. This robust ventral phenotype of Dyrk1B further supports its interference with Shh/Gli signaling.

Indeed, here we provide evidence that Dyrk1B overexpression inhibits Shh signaling, by decreasing Shh, Gli2 and Gli3 mRNA levels. Consistently, we also showed that the pharmacological inhibition of endogenous Dyrk1B, by AZ191 resulted in increased transcription of the Shh, Gli2 and Gli3 genes. Shh signaling is mediated by overlapping but distinct functions of Gli transcription factors. Gli2 and Gli3 are proteolytically processed in the absence of Shh, acting as transcriptional repressors, while Gli1 is not, acting only as transcriptional activator [85–87]. In the ventral neural tube Gli2 is the principal activator of Shh pathway [88–90] and in cooperation with Gli3 regulates ventral patterning and proliferation [91–93]. Our data are in agreement with other studies that have implicated Dyrk1B in canonical and non-canonical Shh pathway in many cancers [50–53]. Particularly, Dyrk1B was found to inhibit canonical Shh and to activate the non-canonical Shh signaling through mTOR/AKT pathway [52]. Moreover, Lauth and colleagues have reported that overexpression of Dyrk1B in NIH3T3 cells blocks SMO-initiated signaling upstream of Suppressor of fused homolog SUFU [50]. In addition, overexpression of Dyrk1B in Panc1 cells enhances the protein stability of GLI1 by preventing its proteasomal degradation [52], while pharmacological inhibition of Dyrk1B results in up-regulation of Shh signaling [52].

In accordance, *in phenotype rescue in ovo* experiments, we demonstrated that activation of the Shh pathway with the use of SMO agonist, SAG, reverses the Dyrk1B effect on ventral phenotypes, by restoring the number of p2, pMN and p3 progenitors. Our data are in agreement with the function of Shh during SC development. Shh initially secreted from NC and later from FP, acts as a long-range graded morphogen that controls the ventral patterning of SC [6–11], as well as cell cycle progression and survival of ventral progenitors [7–11]. Ectopic expression or activation of Shh pathway results in hyper-proliferation of ventral progenitors [10, 11] and conversely inhibition of Shh signaling, results in decreased proliferation and survival of ventral progenitors [10, 94–96]. Thus, the effect of Dyrk1B on cellular proliferation and survival of ventral progenitors p2, pMN and p3 could be also explained by its interference with Shh signaling. In good agreement, our data showed that both SAG and AZ191 are able to activate the Shh pathway, as indicated by enhanced expression of Shh, Gli2 and Gli3, and by increased expression of the early responding proximal Shh targets, FoxA2 and Nkx2.2 [9, 97]. Moreover, these treatments result in hyper-proliferation of p2, pMN and p3 progenitors and in increased number of MNs and of later-formed LMCm MNs. Interestingly, the extensive loss of MNs observed at E4 chick Dyrk1B-electroporated SC, which is selectively reflected at E6 in reduced number of LMCm MNs, could be explained by the fact that Shh is exclusively co-expressed with Dyrk1B, by LMCm MNs at the brachial level of E6 chick SC. Shh exclusive expression by LMCm MNs was reported in a previous study by Nam and colleagues, where Shh was found crucial for LMCm specification [81]. Therefore, our findings are in agreement with these observations [81].

**Fig. 12:**
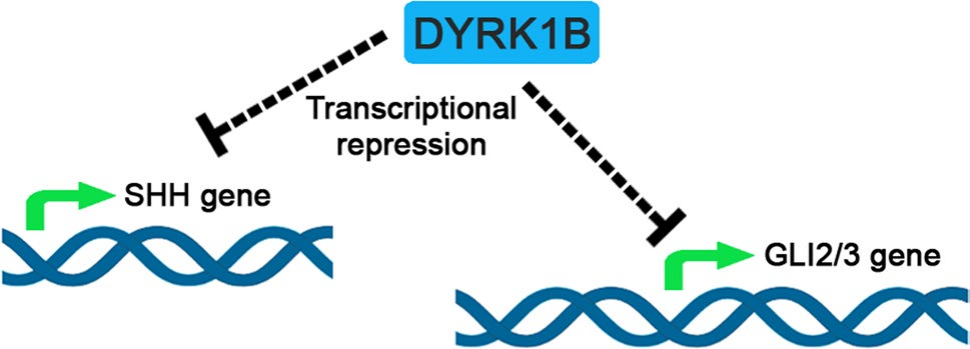
Schematic representation of Dyrk1B effect on Shh, Gli2 and Gli3 transcription. Dyrk1B kinase acts as a potent transcriptional suppressor of Shh, Gli2 and Gli3 gene transcription via an as yet unknown mechanism. Since Dyrk1B is a nuclear kinase, it is likely to act directly or indirectly on the transcriptional machinery of Shh, Gli2 and Gli3 by interacting with transcription or/and chromatin remodeling factors, resulting in transcriptional alterations in gene expression of Shh, Gli2 and Gli3

Our data suggest that Dyrk1B may act as transcriptional repressor of Shh, Gli2 and Gli3 in the embryonic chick SC. Since Dyrk1B is localized in the cell nucleus, it is likely to act on the transcriptional machinery that controls Shh, Gli2 and Gli3 gene expression. Previously, Dyrk1B was found to interact with class II histone deacetylases in skeletal muscle [23]. Notably, it has also been reported that Dyrk1B may directly interact with chromatin modifiers with critical roles in transcriptional regulation, including CBP (CREBBP), p300, p400, KAT8, MSL3, and HDAC5 [98, 99]. A plausible scenario would be that Dyrk1B physically or functionally interacts with transcription factors, such as FoxA2 and possibly Arx for Shh, or chromatin remodeling factors, resulting in transcriptional alterations in gene expression of Shh, Gli2 and Gli3 (Fig. 12). Given that Shh initially secreted from the notochord induces the expression of FoxA2 in the floor plate which in turn induces Shh expression and that of its target gene Nkx2.2 [9, 97], Dyrk1B may interfere at this step of signal transduction. Thus, we envisage a molecular mechanism via which Dyrk1B is recruited over the regulatory DNA sequences of Shh, Gli2 and Gli3 genes, where it modifies the activity of the chromatin-associated complexes to suppress their expression. However, based on our observations, we cannot exclude the possibility of an indirect regulation of these genes by Dyrk1B. Further, we cannot exclude that Dyrk1B has additional non-cell autonomous functions. Alternatively, the effect of Dyrk1B on Gli3 expression may also be mediated through Wnt/β-catenin pathway, as Wnt signaling was found to control Gli3 expression and consequently the cell fate of ventral progenitors [100, 101]. Therefore, the precise mechanism of Dyrk1B function on Shh, Gli2 and Gli3 gene transcription should be further investigated in the future.

In conclusion, we demonstrated that Dyrk1B is a major regulator controlling the balance between cell cycle progression and differentiation during SC development. Moreover, we revealed a novel role for Dyrk1B kinase in the control of the number of ventral progenitor and neuronal subtypes, as well as in the columnar organization of spinal motor neurons via the Sonic hedgehog/Gli pathway.

### Supplementary Information

Below is the link to the electronic supplementary material.

Supplementary file1 (DOCX 21 KB)

Supplementary file2 (TIF 1222 KB) Sequence alignment of 3’-end of Dyrk1B coding region among the species. A cDNA fragment of 253 bp corresponding to 3’-end of coding region of chick Dyrk1B cDNA was cloned by RT-PCR from E4 chick spinal cord. Sequence alignment showed that chick (cDyrk1B) displays 100% similarity with mouse (mDyrk1B), 96.05 % with rat (rDyrk1B) and 90.91% with human (hDyrk1B) homologues, respectively

Supplementary file3 (TIF 9964 KB) Dyrk1B protein expression levels decline during chick and mouse CNS development. A. During CNS development Dyrk1B protein levels are reduced in both developing chick and mouse spinal cord and brain, from embryonic stages E3 to E9 for chick and E12.5 to E14.5 and postnatal stages P0 to P15 for mouse, as revealed by Western blot analysis. B. Quantification and normalization of Dyrk1B protein expression levels is shown relatively to β-tubulin using the ImageJ software. Error bars: SEM

Supplementary file4 (TIF 11622 KB) Dyrk1B, Shh, FoxA2 and Nkx2.2 expression in E9.5 mouse spinal cord. Co-expression of Dyrk1B with Shh and FoxA2 is seen in the notochord (NC) and floor plate (FP) (i-iii), Nkx2.2 at the p3 domain (iv). Scale bars: 100 µm. Spinal cord sections correspond to the forelimb level

Supplementary file5 (TIF 4042 KB) Unilateral Dyrk1B in ovo electroporation. A. Forced expression of Dyrk1B resulted in a 3.0-fold increase of mRNA levels, compared to wild-type SC, as estimated by real time qRT-PCR. B, C. Accordingly, a 2.7-fold increase of Dyrk1B protein was estimated by Western Blot (WB). Quantification and normalization of Dyrk1B protein levels is shown relatively to β-tubulin using the ImageJ software. Data were obtained from one experiment. Total mRNA and protein lysates were derived from of a pool of 3 embryonic chick spinal cords in each case

Supplementary file6 (TIF 72 KB) Dyrk1B overexpression at E2 chick spinal cord does not affect the dorsal patterning. Dyrk1B overexpression did not affect the normalized number of dorsal Islet1^+^ dI3 neurons over total nuclei (indicated by an asterisk in Fig.3A), when compared spinal cord contralaterally in Dyrk1B-GFP-electroporated embryos. Also, no differences were observed contralaterally in GFP-electroporated control embryos (p>0.05, n=12 sections from 4 embryos). Data are mean ± SEM, *p≤0.05, **p≤0.01, ***p≤0.001; ns, non-significant (two-tailed Unequal variance t-test)

Supplementary file7 (TIF 175 KB) AZ191 increases the number of p2, pMN and p3 ventral progenitors at E4. AZ191 administration at E2 increases the normalized number to total nuclei of A. p2 progenitors (Nkx6.1^+^/Olig2^-^) by 32.57% ± 9.86 (p≤0.01, n=23 sections from 4 embryos), B. pMN progenitors (Nkx6.1^+^/Olig2^+^) by 31.90% ± 4.89 (p≤0.001, n=23 sections from 4 embryos) and C. p3 progenitors (Nkx6.1^+^/Olig2^-^) by 61.25% ± 23.75 (p≤0.05, n=23 sections from 4 embryos), as compared to DMSO-treated embryos. Data are mean ± SEM, *p≤0.05, **p≤0.01, ***p≤0.001; ns, non-significant (two-tailed Unequal variance t-test)

Supplementary file8 (TIF 6476 KB) Dyrk1B activity affects the ratio of Gli3A/Gli3R and the expression of FoxA2 and Nkx2.2 transcription factors. Western blot analysis and quantification in E4 SC protein lysates derived from pools of 3 chick embryos corresponding to each experimental condition, as indicated. A. The Gli3A/Gli3R ratio, was estimated after normalization of each Gli3 form to β-tubulin and all cases were compared to wt. Dyrk1B overexpression results in a reduced ratio of Gli3A/Gli3R by 1.20-fold, while in AZ191 and SAG-treated embryos, an increased ratio of 1.57-fold and 1.74-fold was respectively observed. In Dyrk1B/GFP-electroporated embryos treated with AZ191 the Gli3A/Gli3R ratio was practically restored to the levels of wt and GFP-electroporated embryos. B. Dyrk1B overexpression reduces FoxA2 levels, normalized to GAPDH, by 1.32-fold, while in AZ191 and SAG-treated embryos FoxA2 expression is increased by 1.55-fold and by 2.86-fold respectively, all compared to wt. In Dyrk1B/GFP-electroporated embryos treated with AZ191, FoxA2 expression was restored to the levels of control embryos. C. Dyrk1B overexpression reduces Nkx2.2 protein levels, normalized to GAPDH, by 1.50-fold, while in AZ191 and SAG-treated embryos Nkx2.2 is increased by 1.33-fold and by 2.10-fold respectively, all compared to wt. In Dyrk1B/GFP-electroporated embryos treated with AZ191, Nkx2.2 expression was similar to the levels of wt and GFP-electroporated embryos. Data were obtained from one experiment. Quantification and normalization of expression levels was performed using the ImageJ software

Supplementary file9 (TIF 13225 KB) AZ191 and SAG administration did not affect MMC and LMCl MNs at E6 chick spinal cord. A, B. Inhibition of endogenous Dyrk1B activity by AZ191 administration at E2 (LOF), does not affect the number of either MMC (Lim3^+^) MNs (Ai, iii) (p>0.05, n=19 sections from 4 embryos), or the number of LMCl (HB9^+^/Foxp1^+^) MNs (Aii, iv) (p>0.05, n=19 sections from 4 embryos) at E6, as compared with wild-type embryos. C, D. Similarly, no effect was observed, when the Shh pathway was activated by administration of SAG at E2, in the number and columnar organization of MMC (Lim3^+^) MNs (Ci, iii) (p>0.05, n=19 sections from 4 embryos), or the number of LMCl (HB9^+^/Foxp1^+^) MNs (Cii, iv) (p>0.05, n=19 from 4 embryos) at E6. Cell numbers are normalized to total cell nuclei. Scale bars: 100 µm. Data are mean ± SEM, *p≤0.05, **p≤0.01, ***p≤0.001; ns, non-significant (two-tailed Unequal variance t-test). Spinal cord sections correspond to the brachial level

## Data Availability

All relevant data of this study are available within the article and its Supplementary Information files or from corresponding author on request.

## Abbreviations

Shh: Sonic Hedgehog
SMO: Smoothened
SAG: Smoothened agonist
NC: Notochord
FP: Floor plate
SC: Spinal cord
MNs: Motor neurons
INs: Interneurons
SpMNs: Spinal motor neurons
MN domain: Motor neuron domain
pMNs: Motor neurons progenitors
VZ: Ventricular zone
MZ: Mantle zone
CC: Central canal
NPCs: Neural progenitor cells
CNS: Central nervous system
M&M: Materials and methods
