# Supplementary material for: Mirk/Dyrk1B controls ventral spinal cord development via Shh pathway
Source: Cell Mol Life Sci. 2024 Jan 31;81(1):70. doi: 10.1007/s00018-023-05097-9 (PMC10830675; doi:10.1007/s00018-023-05097-9)
Supplement: Supplementary file 1 — Supplementary file1 (DOCX 21 KB) [file 18_2023_5097_MOESM1_ESM.docx]

| **Supplementary Table 1. Primary antibodies used in the current study** | | | | | | |
| --- | --- | --- | --- | --- | --- | --- |
| **No** | **Primary antibody name** | **Host & Clonality** | **Appilcation & Dilution** | **Manufacturer** | **Cat#** | **RRID** |
| 1 | β-tubulin (H-235) | rabbit polyclonal | WB/ 1:1000 | Santa Cruz  Biotechnology | sc-9104 | AB_2241191 |
| 2 | βIII-tubulin (TuJ1 clone) | mouse monoclonal | IHC/1:500 | BioLegend | 801202 | AB_10063408 |
| 3 | 5’Bromo-2’deoxyuridine (BrdU) (Bu20A) | mouse monoclonal | IHC/1:200 | Santa Cruz Biotechnology | sc-20045 | AB_626767 |
| 4 | Chx10 | sheep polyclonal | IHC/1:250 | Millipore | AB9016 | AB_2216009 |
| 5 | Cleaved Caspase 3 (Asp175) (Casp3) | rabbit polyclonal | IHC/1:200 | Cell Signaling Technology | 9661 | AB_2341188 |
| 6 | Digoxigenin (DIG) alkaline phosphatase (AP) conjugate | sheep polyclonal | ISH/1:1000 | Roche | 11093274910 | AB_514497 |
| 7 | Doublecortin (DCX) (N-19) | goat polyclonal | IHC/1:50 | Santa Cruz Biotechnology | sc-8067 | AB_2088491 |
| 8 | Dyrk1B | rabbit monoclonal | WB/ 1:1000  IHC/1:100 | Abcam | ab124960 | AB_10972162 |
| 9 | FOXA2 [HNF-3β (M-20)] | mouse monoclonal | WB/ 1:500 | Santa Cruz  Biotechnology | sc-365062 | AB_1124660 |
| 10 | FOXA2 [HNF-3β (M-20)] | goat polyclonal | IHC/1:50 | Santa Cruz  Biotechnology | sc-6554 | AB_2262810 |
| 11 | FoxP1 (C-terminal) | rabbit polyclonal | IHC/1:100 | Abcam | ab227788 | --- |
| 12 | GAPDH (G-9) | mouse monoclonal | WB/ 1:1000 | Santa Cruz  Biotechnology | sc-365062 | AB_10847862 |
| 13 | GFP | chicken polyclonal | IHC/1:500 | Thermo Fisher Scientific | A10262 | AB_2534023 |
| 14 | GFP | chicken polyclonal | IHC/1:600 | Abcam | ab13970 | AB_300798 |
| 15 | GLI3 | rabbit polyclonal | WB/ 1:1000 | Novus Biologicals | NBP2-29627 | --- |
| 16 | HB9/MNR2 | mouse monoclonal | IHC/1:50 | DSHB | 81.5C10 | AB_2145209 |
| 17 | Islet1/2 | mouse monoclonal | IHC/1:50 | DSHB | 39.4D5 | AB_2314683 |
| 18 | Lim3 (Lhx3) | rabbit polyclonal | IHC/1:250 | Thermo Fisher Scientific | PA1-29491 | AB_2135675 |
| 19 | Nkx2.2 | rabbit polyclonal | WB/ 1:1000 | Abcam | ab272915 | --- |
| 20 | Nkx2.2 | mouse monoclonal | IHC/1:50 | DSHB | 74.5A5 | AB_531794 |
| 21 | Nk­x6.1 | mouse monoclonal | IHC/1:50 | DSHB | F55A10 | AB_532378 |
| 22 | Olig2 | rabbit polyclonal | IHC/1:200 | Millipore | AB9610 | AB_570666 |
| 23 | Pax3 | mouse monoclonal | IHC/1:50 | DSHB | --- | AB_528426 |
| 24 | phospho-Histone H3 (Ser10) (PH3) | rabbit polyclonal | IHC/1:200 | Millipore | 06-570 | AB_310177 |
| 25 | Shh | mouse monoclonal | IHC/1:50 | DSHB | 5E1 | AB_528466 |
| 26 | Sox2 (Y-17) | goat polyclonal | IHC/1:200 | Santa Cruz Biotechnology | sc-17320 | AB_2286684 |

| **Supplementary Table 2. Secondary antibodies used in the current study** | | | | | | |
| --- | --- | --- | --- | --- | --- | --- |
| **No** | **Secondary antibody name/conjucate** | **Host & Clonality** | **Appilcation & Dilution** | **Manufacturer** | **Cat#** | **RRID** |
| 1 | anti-chicken  Alexa-Fluor 488 | donkey polyclonal | IHC/ 1:500 | Jackson ImmunoResearch Labs | 703-545-155 | AB_2340375 |
| 2 | anti-rabbit  CF488A | donkey polyclonal | IHC/ 1:500 | Biotium | 20015 | AB_10559669 |
| 3 | anti-rabbit  CF555 | donkey polyclonal | IHC/ 1:500 | Biotium | 20038 | AB_10558011 |
| 4 | anti-rabbit  Alexa-Fluor 647 | donkey polyclonal | IHC/ 1:500 | Thermo Fisher Scientific | A-31573 | AB_2536183 |
| 5 | anti-mouse  CF488A | donkey polyclonal | IHC/ 1:500 | Biotium | 20014 | AB_10561327 |
| 6 | anti-mouse  CF543 | rabbit polyclonal | IHC/ 1:500 | Biotium | 20305 | AB_2923245 |
| 7 | anti-mouse  Alexa-Fluor 647 | mouse monoclonal | IHC/ 1:500 | Thermo Fisher Scientific | A-31571 | AB_162542 |
| 9 | anti-goat  Alexa-Fluor 546 | donkey polyclonal | IHC/ 1:500 | Thermo Fisher Scientific | A-11056 | AB_2534103 |
| 10 | anti-goat  Alexa-Fluor 647 | donkey polyclonal | IHC/ 1:500 | Thermo Fisher Scientific | A-21447 | AB_2535864 |
| 11 | anti-Sheep  Cy3 | donkey polyclonal | IHC/ 1:500 | Jackson ImmunoResearch Labs | 713-165-147 | AB_2315778 |
| 12 | anti-rabbit  HRP | donkey polyclonal | WB/1:5000 | Chemicon | AP182P | AB_92591 |
| 13 | anti-mouse  HRP | donkey polyclonal | WB/1:2000 | Chemicon | AP192P | AB_92658 |
