# Supplementary figures and images for: Mirk/Dyrk1B controls ventral spinal cord development via Shh pathway
Source: Cell Mol Life Sci. 2024 Jan 31;81(1):70. doi: 10.1007/s00018-023-05097-9 (PMC10830675; doi:10.1007/s00018-023-05097-9)

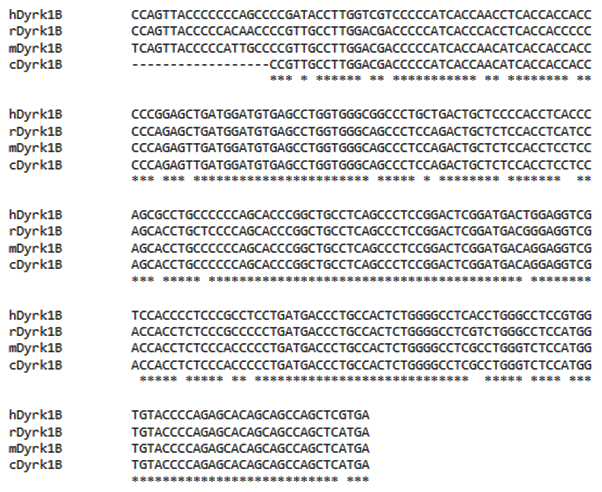

Supplement: Supplementary file 2 — Supplementary file2 (TIF 1222 KB) Sequence alignment of 3’-end of Dyrk1B coding region among the species. A cDNA fragment of 253 bp corresponding to 3’-end of coding region of chick Dyrk1B cDNA was cloned by RT-PCR from E4 chick spinal cord. Sequence alignment showed that chick (cDyrk1B) displays 100% similarity with mouse (mDyrk1B), 96.05 % with rat (rDyrk1B) and 90.91% with human (hDyrk1B) homologues, respectively [file 18_2023_5097_MOESM2_ESM.tif]

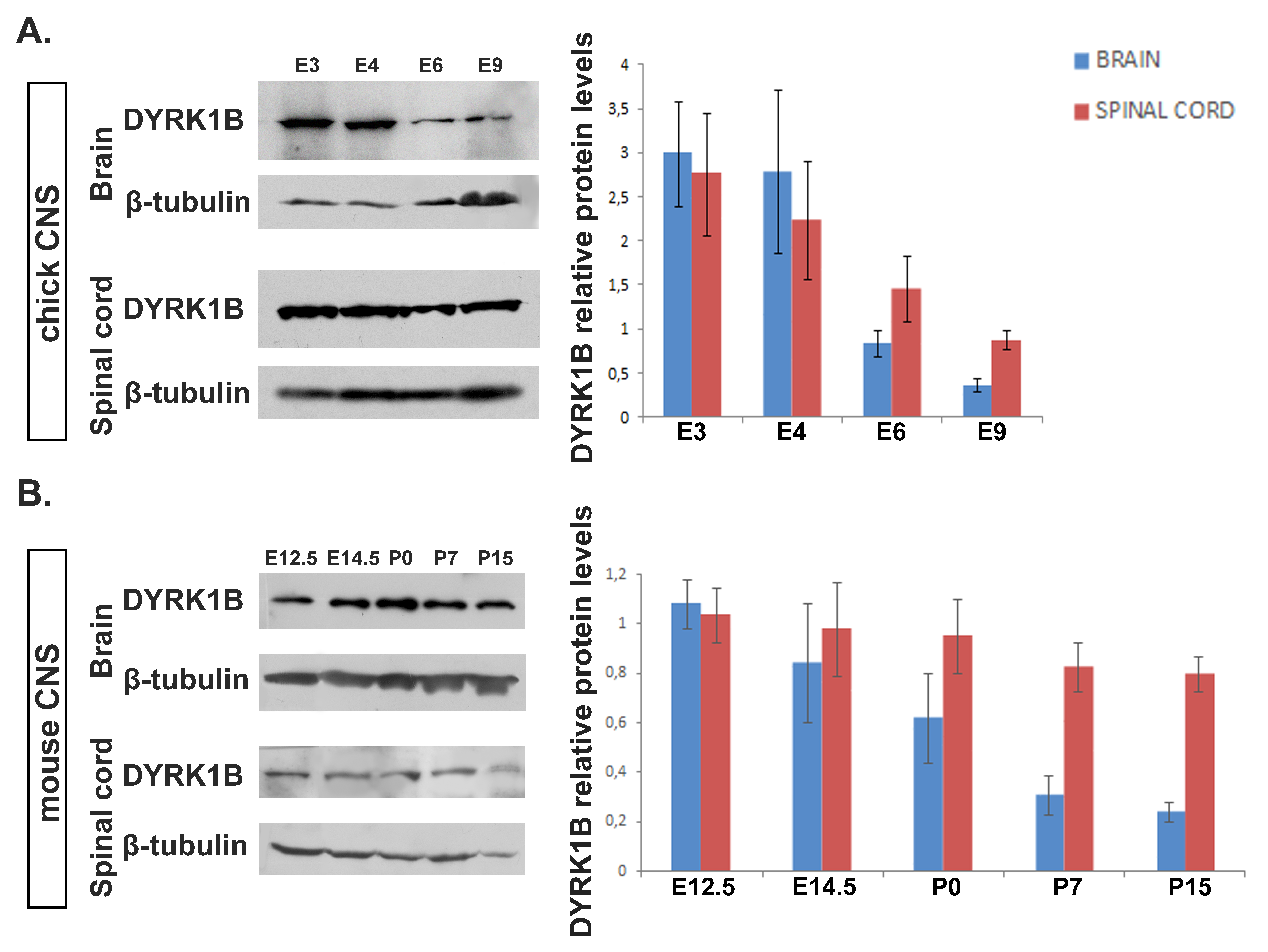

Supplement: Supplementary file 3 — Supplementary file3 (TIF 9964 KB) Dyrk1B protein expression levels decline during chick and mouse CNS development. A. During CNS development Dyrk1B protein levels are reduced in both developing chick and mouse spinal cord and brain, from embryonic stages E3 to E9 for chick and E12.5 to E14.5 and postnatal stages P0 to P15 for mouse, as revealed by Western blot analysis. B. Quantification and normalization of Dyrk1B protein expression levels is shown relatively to β-tubulin using the ImageJ software. Error bars: SEM [file 18_2023_5097_MOESM3_ESM.tif]

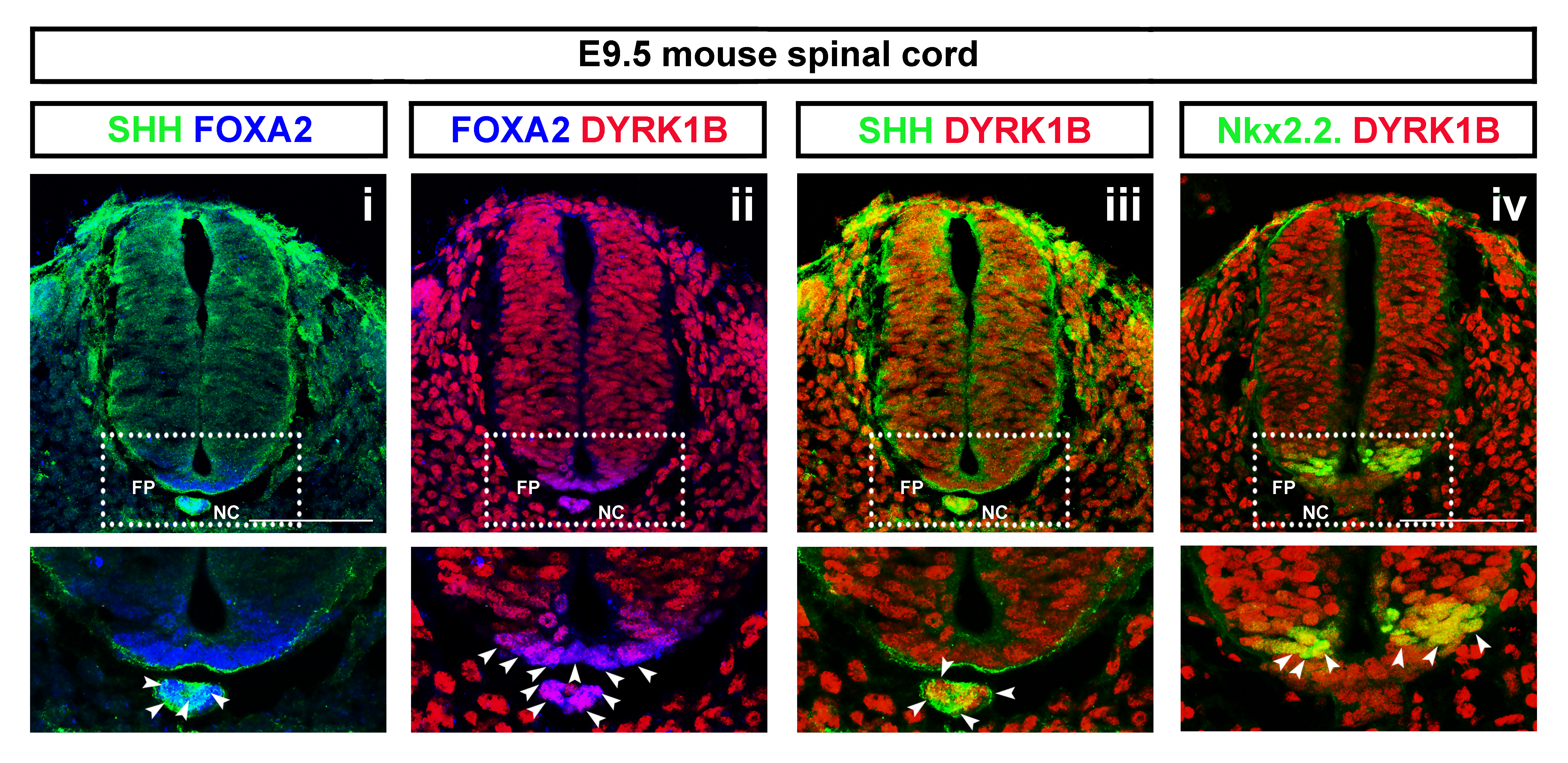

Supplement: Supplementary file 4 — Supplementary file4 (TIF 11622 KB) Dyrk1B, Shh, FoxA2 and Nkx2.2 expression in E9.5 mouse spinal cord. Co-expression of Dyrk1B with Shh and FoxA2 is seen in the notochord (NC) and floor plate (FP) (i-iii), Nkx2.2 at the p3 domain (iv). Scale bars: 100 µm. Spinal cord sections correspond to the forelimb level [file 18_2023_5097_MOESM4_ESM.tif]

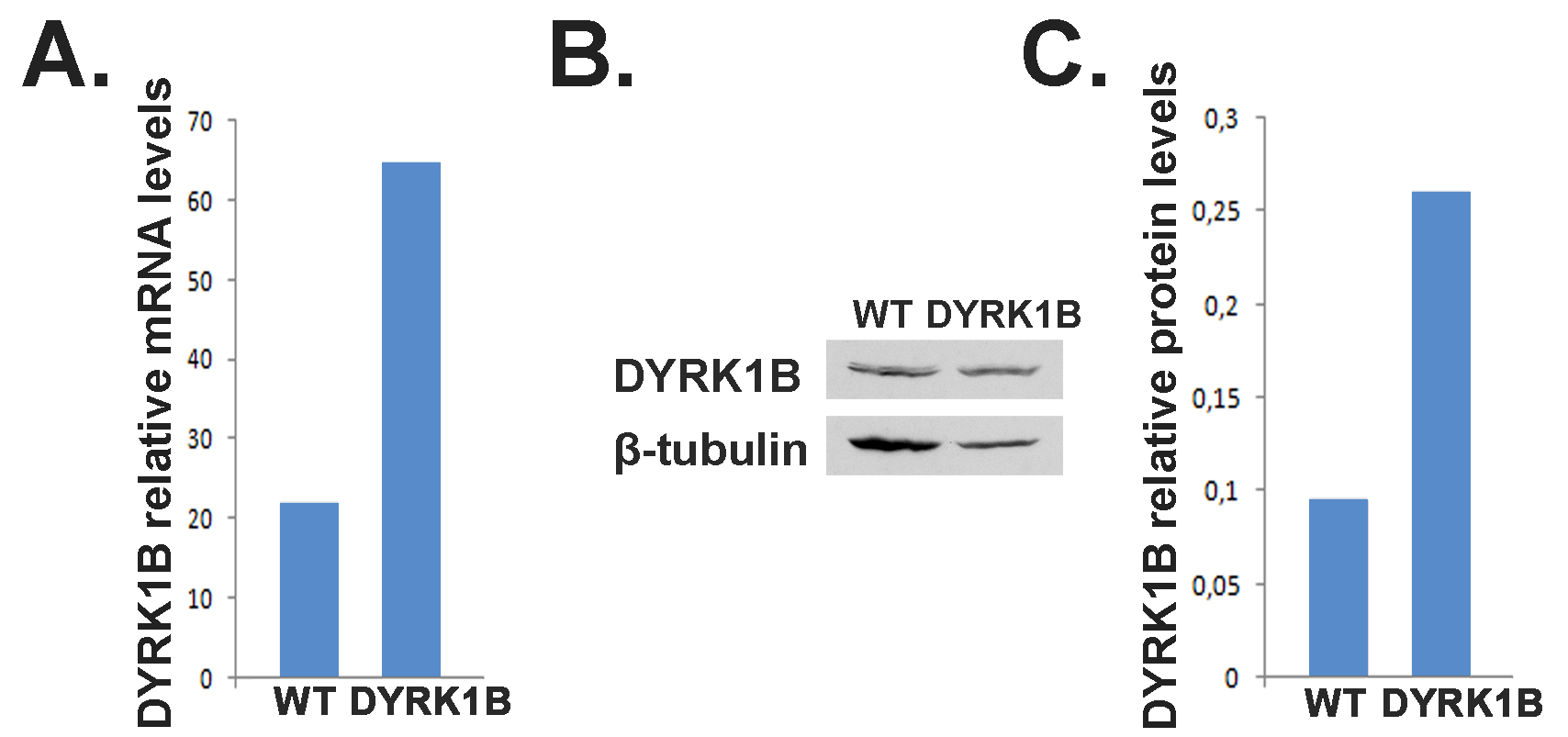

Supplement: Supplementary file 5 — Supplementary file5 (TIF 4042 KB) Unilateral Dyrk1B in ovo electroporation. A. Forced expression of Dyrk1B resulted in a 3.0-fold increase of mRNA levels, compared to wild-type SC, as estimated by real time qRT-PCR. B, C. Accordingly, a 2.7-fold increase of Dyrk1B protein was estimated by Western Blot (WB). Quantification and normalization of Dyrk1B protein levels is shown relatively to β-tubulin using the ImageJ software. Data were obtained from one experiment. Total mRNA and protein lysates were derived from of a pool of 3 embryonic chick spinal cords in each case [file 18_2023_5097_MOESM5_ESM.tif]

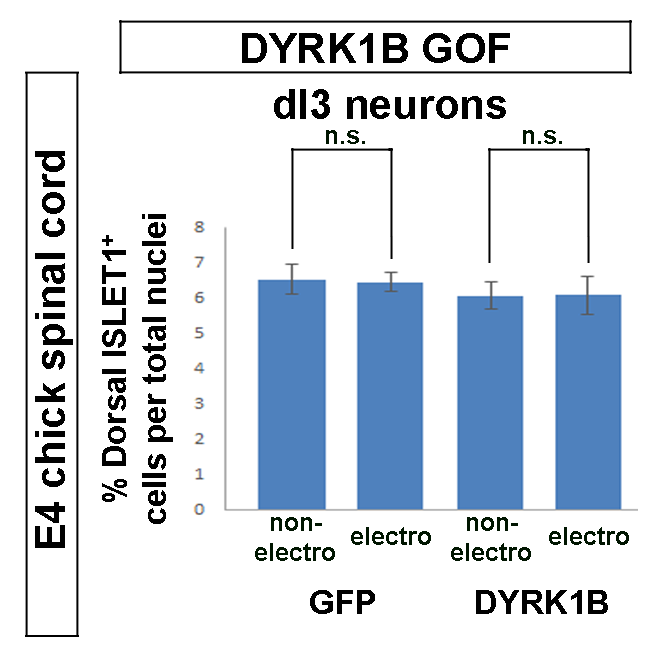

Supplement: Supplementary file 6 — Supplementary file6 (TIF 72 KB) Dyrk1B overexpression at E2 chick spinal cord does not affect the dorsal patterning. Dyrk1B overexpression did not affect the normalized number of dorsal Islet1+ dI3 neurons over total nuclei (indicated by an asterisk in Fig.3A), when compared spinal cord contralaterally in Dyrk1B-GFP-electroporated embryos. Also, no differences were observed contralaterally in GFP-electroporated control embryos (p>0.05, n=12 sections from 4 embryos). Data are mean ± SEM, *p≤0.05, **p≤0.01, ***p≤0.001; ns, non-significant (two-tailed Unequal variance t-test) [file 18_2023_5097_MOESM6_ESM.tif]

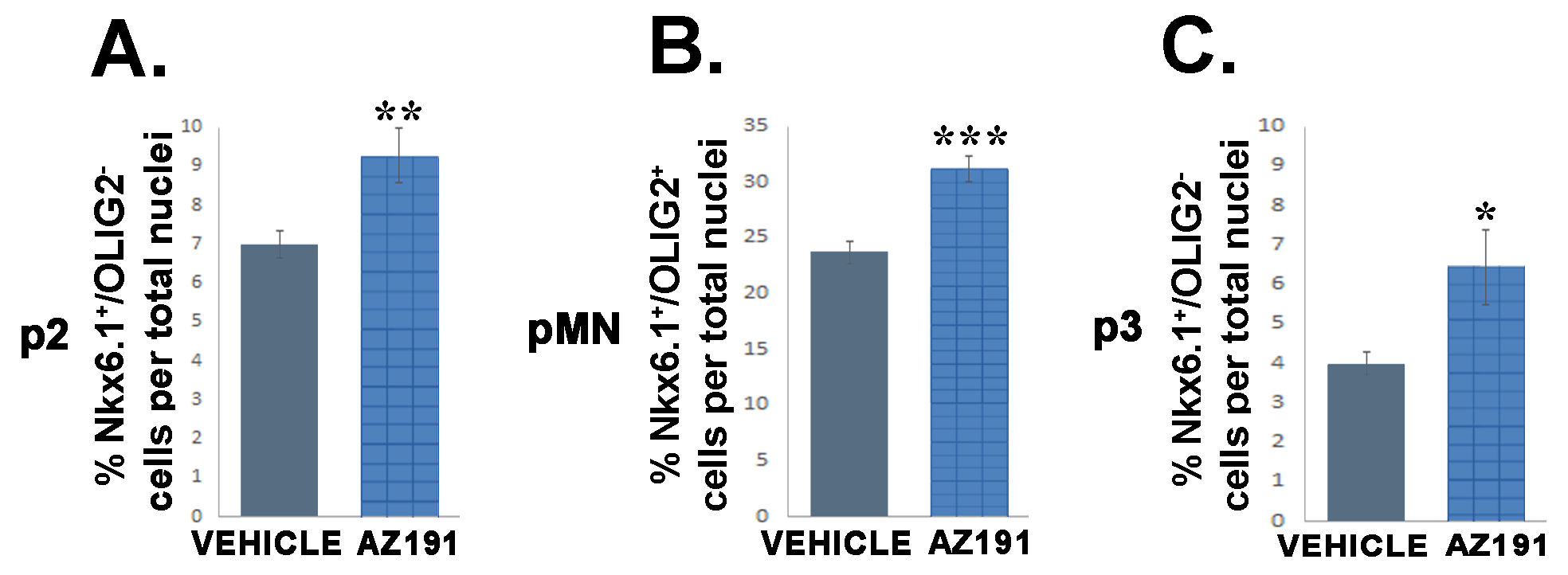

Supplement: Supplementary file 7 — Supplementary file7 (TIF 175 KB) AZ191 increases the number of p2, pMN and p3 ventral progenitors at E4. AZ191 administration at E2 increases the normalized number to total nuclei of A. p2 progenitors (Nkx6.1+/Olig2-) by 32.57% ± 9.86 (p≤0.01, n=23 sections from 4 embryos), B. pMN progenitors (Nkx6.1+/Olig2+) by 31.90% ± 4.89 (p≤0.001, n=23 sections from 4 embryos) and C. p3 progenitors (Nkx6.1+/Olig2-) by 61.25% ± 23.75 (p≤0.05, n=23 sections from 4 embryos), as compared to DMSO-treated embryos. Data are mean ± SEM, *p≤0.05, **p≤0.01, ***p≤0.001; ns, non-significant (two-tailed Unequal variance t-test) [file 18_2023_5097_MOESM7_ESM.tif]

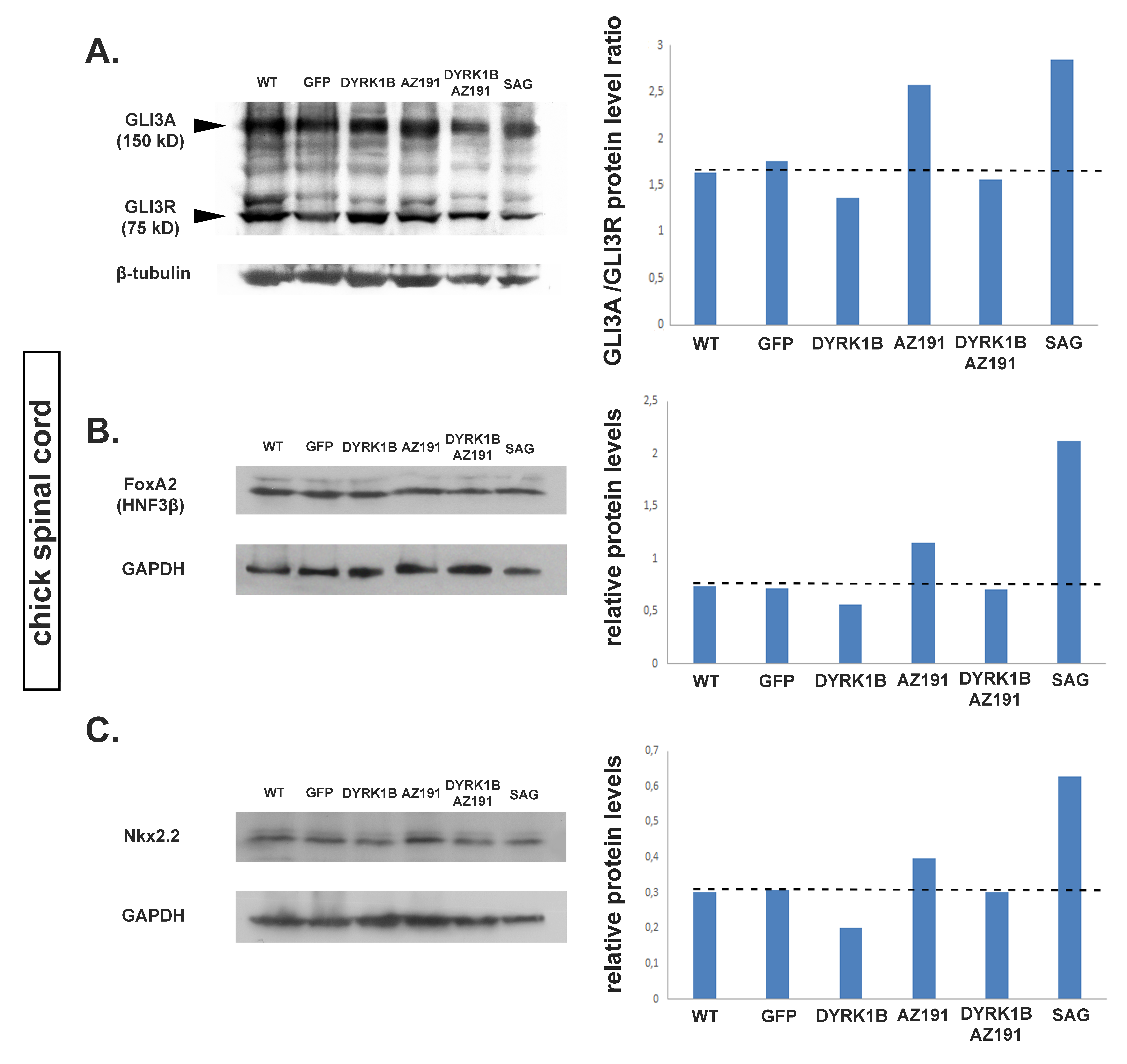

Supplement: Supplementary file 8 — Supplementary file8 (TIF 6476 KB) Dyrk1B activity affects the ratio of Gli3A/Gli3R and the expression of FoxA2 and Nkx2.2 transcription factors. Western blot analysis and quantification in E4 SC protein lysates derived from pools of 3 chick embryos corresponding to each experimental condition, as indicated. A. The Gli3A/Gli3R ratio, was estimated after normalization of each Gli3 form to β-tubulin and all cases were compared to wt. Dyrk1B overexpression results in a reduced ratio of Gli3A/Gli3R by 1.20-fold, while in AZ191 and SAG-treated embryos, an increased ratio of 1.57-fold and 1.74-fold was respectively observed. In Dyrk1B/GFP-electroporated embryos treated with AZ191 the Gli3A/Gli3R ratio was practically restored to the levels of wt and GFP-electroporated embryos. B. Dyrk1B overexpression reduces FoxA2 levels, normalized to GAPDH, by 1.32-fold, while in AZ191 and SAG-treated embryos FoxA2 expression is increased by 1.55-fold and by 2.86-fold respectively, all compared to wt. In Dyrk1B/GFP-electroporated embryos treated with AZ191, FoxA2 expression was restored to the levels of control embryos. C. Dyrk1B overexpression reduces Nkx2.2 protein levels, normalized to GAPDH, by 1.50-fold, while in AZ191 and SAG-treated embryos Nkx2.2 is increased by 1.33-fold and by 2.10-fold respectively, all compared to wt. In Dyrk1B/GFP-electroporated embryos treated with AZ191, Nkx2.2 expression was similar to the levels of wt and GFP-electroporated embryos. Data were obtained from one experiment. Quantification and normalization of expression levels was performed using the ImageJ software [file 18_2023_5097_MOESM8_ESM.tif]

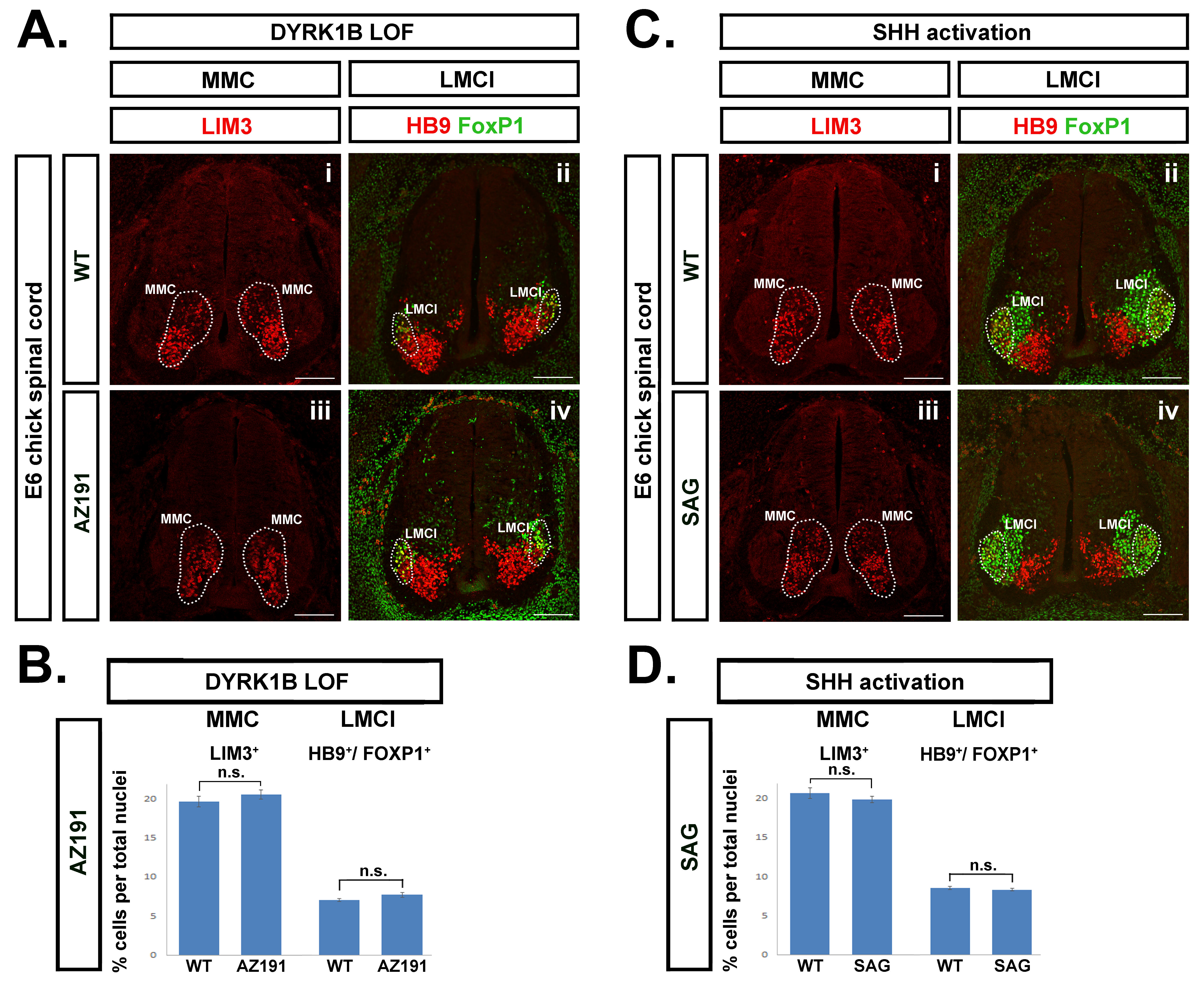

Supplement: Supplementary file 9 — Supplementary file9 (TIF 13225 KB) AZ191 and SAG administration did not affect MMC and LMCl MNs at E6 chick spinal cord. A, B. Inhibition of endogenous Dyrk1B activity by AZ191 administration at E2 (LOF), does not affect the number of either MMC (Lim3+) MNs (Ai, iii) (p>0.05, n=19 sections from 4 embryos), or the number of LMCl (HB9+/Foxp1+) MNs (Aii, iv) (p>0.05, n=19 sections from 4 embryos) at E6, as compared with wild-type embryos. C, D. Similarly, no effect was observed, when the Shh pathway was activated by administration of SAG at E2, in the number and columnar organization of MMC (Lim3+) MNs (Ci, iii) (p>0.05, n=19 sections from 4 embryos), or the number of LMCl (HB9+/Foxp1+) MNs (Cii, iv) (p>0.05, n=19 from 4 embryos) at E6. Cell numbers are normalized to total cell nuclei. Scale bars: 100 µm. Data are mean ± SEM, *p≤0.05, **p≤0.01, ***p≤0.001; ns, non-significant (two-tailed Unequal variance t-test). Spinal cord sections correspond to the brachial level [file 18_2023_5097_MOESM9_ESM.tif]
